# Preparation of Templated Materials and Their Application to Typical Pollutants in Wastewater: A Review

**DOI:** 10.3389/fchem.2022.882876

**Published:** 2022-04-05

**Authors:** Hanbing Li, Li Wang, Yifei Wei, Wei Yan, Jiangtao Feng

**Affiliations:** Xi’an Key Laboratory of Solid Waste Recycling and Resource Recovery, Department of Environmental Science and Engineering, School of Energy and Power Engineering, Xi’an Jiaotong University, Xi’an, China

**Keywords:** template method, moderating behavior, heavy metals, dyes, adsorption mechanism

## Abstract

As the pollution and destruction of global water resources become more and more severe, the treatment of wastewater has attracted significant attention. The template method is a synthetic method in which the template is the main configuration to control, influence, and modify the morphology as well as control the dimensions of the material, thus achieving the properties that determine the material. It is simple, highly reproducible, and predictable, and more importantly, it can effectively control the pore structure, size, and morphology of the material, providing a novel platform for the preparation of adsorbent materials with excellent adsorption properties. This review focuses on the classification of the templates according to their properties and spatial domain-limiting capabilities, reviews the types of hard and soft template materials and their synthetic routes, and further discusses the modulation of the morphological structure of the materials by the introduction of templates. In addition, the application and adsorption mechanisms of heavy metal ions and dyes are reviewed based on the regulatory behavior of the template method.

## 1 Introduction

Pollution (Yang et al., 2019; Zhang et al., 2019; Zhao et al., 2019), especially water pollution, has been a global problem that needs to be addressed immediately , with the rapid development of industrial economy. Human production and living activities result in a considerable discharge of pollutant into the water resources, particularly organic dyes and heavy metals, which can pose a serious threat to people’s living environment if not properly treated. Dyes are extremely harmful to ecosystems due to their toxicity and the reduction of dissolved oxygen in water, while heavy metals can cause pernicious health effects on humans and other organisms (Zare et al., 2018). Therefore, there is an urgent need to develop remediation strategies that effectively mitigate organic dyes and heavy metal pollution.

Nowadays, several techniques have been successfully used for the purification of heavy metal ions and organic dyes in wastewater, such as chemical precipitation, ion exchange, and adsorption. Among them, adsorption has become one of the most influential and promising methods (Zhou et al., 2018) due to its simplicity, low cost, mature process, and low energy consumption (Liu J. et al., 2016; Yu et al., 2018). At present, various adsorbents such as activated carbon (Ekinci et al., 2002; Zhou et al., 2015; Kuroki et al., 2019), zeolites (Briao et al., 2017; Zanin et al., 2017), nanomaterials (Sarma et al., 2019; Madima et al., 2020; Yu et al., 2021), biochar (Wang et al., 2019a; Wang et al., 2019b; Wang L. et al., 2020; Wang et al., 2021; Wei et al., 2022), and metal organic framework polymers (Huang et al., 2018; Pi et al., 2018), have been developed and widely used for the adsorption of wastewater. However, most adsorbents still have some problems, such as uneven pore size distribution and difficulty in achieving effective control of pore size, which directly affect the adsorption capacity of the adsorbent. The template method, which has been developed in recent years, is simple, highly reproducible, and predictable (Liu et al., 2019; Ma et al., 2019). More importantly, the template method enables effective control of the pore structure, dimensions, and morphology of the composite material (Marrakchi et al., 2017), which is expected to be a solution to these problems and achieve efficient adsorption of adsorbents.

The template method, essentially a forming or casting technique, which is different from the conventional methods, in that it needs to introduce a template agent in advance. Whether the reaction takes place in the liquid or solid phase, the template method can effectively modulate the size, morphology, structure, and even the arrangement of the sample. The morphology of the product varies depending on the template chosen, so it is important to select the right template (Liu et al., 2013). The template method is now widely used in water treatment [such as photocatalysis (Ahmad et al., 2017; Yang et al., 2021), adsorption (Libbrecht et al., 2017; Zhao et al., 2020a), and electrochemistry, as well as biology and medicine (Miao et al., 2010; Wu and Xu, 2010; Li et al., 2013)]. This study outlines the classification of the template method, focuses on the hard and soft template methods, discusses the modulating behavior of the template method on the morphological structure of materials, and further discusses the current status of the application of the template method for heavy metal ions and organic dyes in water treatment.

## 2 Preparation and Classification of the Template Method

The template method is based on the morphological characteristics of the synthetic material, introducing its self-assembly or organic molecular system as a template, and effectively regulating and modifying the morphology, structure, and dimensions of the material through forces like hydrogen bonding and Van der Waals forces. It is essentially a forming or casting technique (Thomas et al., 2008), generally speaking, synthesis of the template method includes three main steps (Knezevic et al., 2018): 1) preparation of templates; 2) synthesis of target materials using the templates; and 3) removal of templates.

**FIGURE 1:**
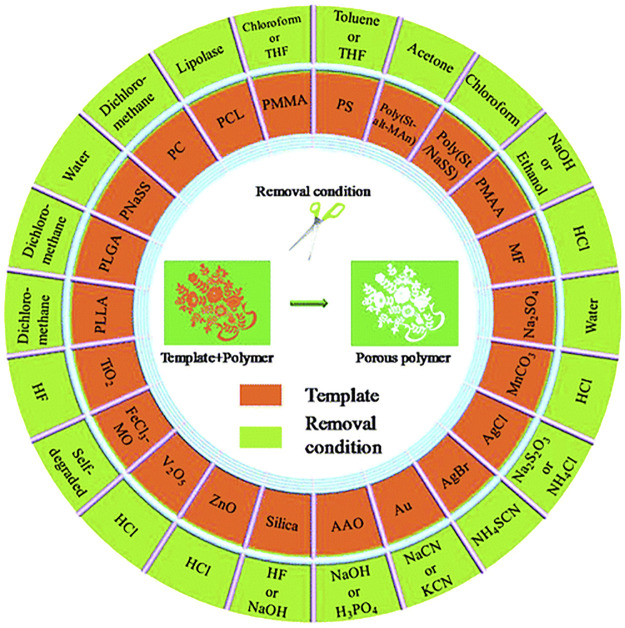
Commonly used polymer templates and their removal conditions (Wu et al., 2012).

In general, there are four requirements for the preparation of template method : 1) in order to achieve successful replication of the template, the surface properties of the template used should be consistent with the raw material; 2) the template should have a well-defined structure, such as an easily adjustable morphology; 3) the template should be easy to remove. Figure 1 summarizes the commonly used polymer templates and their removal conditions (Wu et al., 2012), such as silica templates, as one of the most commonly used templates, are usually removed using hydrofluoric acid or sodium hydroxide; 4) the polymer template should be robust enough to prevent deformation of the pores after template removal.

**FIGURE 2:**
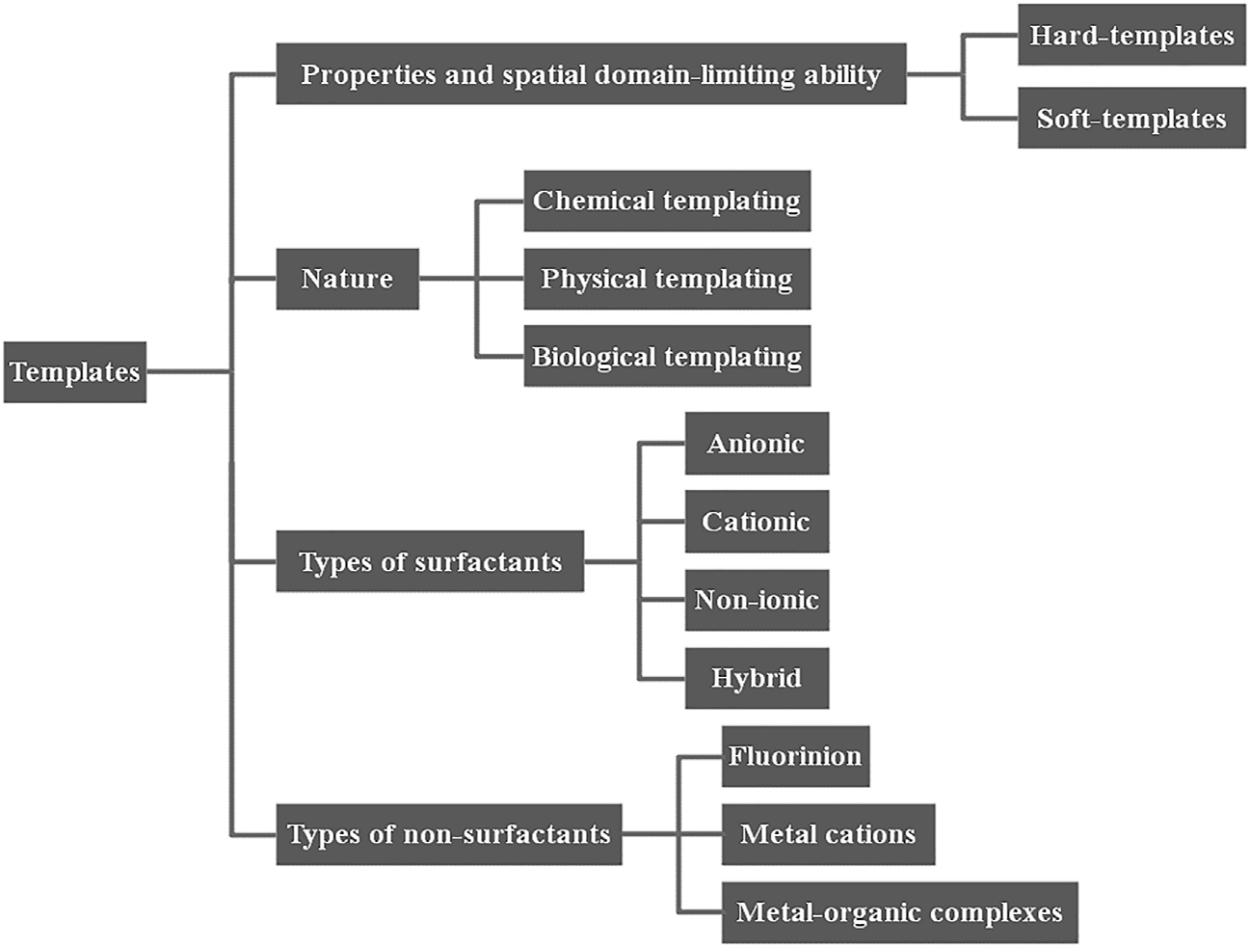
Classifications of templates.

At present, there are various classifications of template agents; the specific classification is shown in Figure 2. One kind of classification can be divided into hard templates (porous silica, silica) and soft templates (biomolecules, surfactants) according to the templates’ own properties and spatial domain-limiting ability (Bao et al., 2016). Depending on the nature of template, it can be subdivided into physical templates, chemical templates, and biological templates (Liu et al., 2013; Zhao et al., 2020b). In addition, some scholars classify it according to surfactants and non-surfactants. The former includes anionic template agents, cationic template agents, non-ionic template agents, and hybrid template agents; while the latter includes metal cations, organic molecules, metal–organic complexes, etc. (Figure 2).

Currently, based on different compositions and characteristics of templates, the most common way to classify them is to divide into hard and soft templates, both of which provide a tunable reaction space for the adsorbent. Among them, hard template is mainly selected from material that is prepared already, such as mesoporous carbon (Libbrecht et al., 2015), mesoporous silica (Marciniak et al., 2019), metal oxides (MO) (El-Hankari et al., 2016), etc. During the preparation process, the raw material is adsorbed or permeated on the surface or internal lattice of the template, and the template is removed after the reaction (Petkovich and Stein, 2013). Materials prepared by the hard template method can be precisely regulated in terms of size and structure, but the removal of the template can easily cause structural damage to the raw material (Liu D. et al., 2016). In contrast, soft template is formed during the reaction, that is, a phase interface is formed between the soft template [such as polymers (Liu D. et al., 2016), biological macromolecules (Zhao et al., 2017), and surfactants (Cao et al., 2014)]; the target product by micelles and a certain spatial structure is established by the internal polymerization of the molecules. In comparison, soft template is easier to remove and the production condition is mild and simple, which is a current trend in the preparation of materials by the template method (Xie et al., 2016). The detailed characteristics and preparation of each of the two template methods are described below.

### 2.1 Hard Template

Hard templates are usually connected by covalent bonds, and the reaction cavity is in dynamic equilibrium, with substances diffusing in and out through the cavity walls, such as molecular sieves, metals (Wang H. et al., 2016), and carbons (Xie et al., 2018). Generally, the preparation process for materials prepared using hard templates is to wrap or infiltrate the selected template with a precursor, transforming the precursors into solid material by chemical or thermal treatment, and then removing the template by chemical etching or high temperature calcination (Petkovich and Stein, 2013).

**FIGURE 3:**
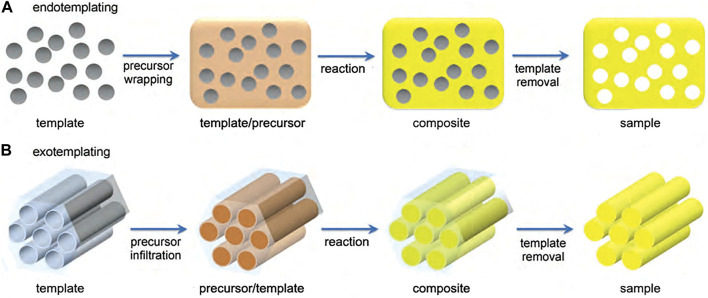
Synthetic mechanism of the hard template method (Wang Y. et al., 2016).

Depending on the inclusion relationship between the hard template and the precursor, the hard template method can be divided into exotemplating and endotemplating methods (Schuth, 2003). As shown in Figure 3A (Wang Y. et al., 2016), endotemplate is the precursor wrapping template. Template molecules enter the precursor and create pore channels, and after the removal of the template, a pore system is formed in the precursor. Exotemplating is the opposite, as shown in Figure 3B, where the precursor penetrates the template. Exotemplate can be materials with structural pores, and the precursor infiltrates into the pore channel. Depending on the connectivity of the template, a porous or finely split material remains after the template removal.

**FIGURE 4:**
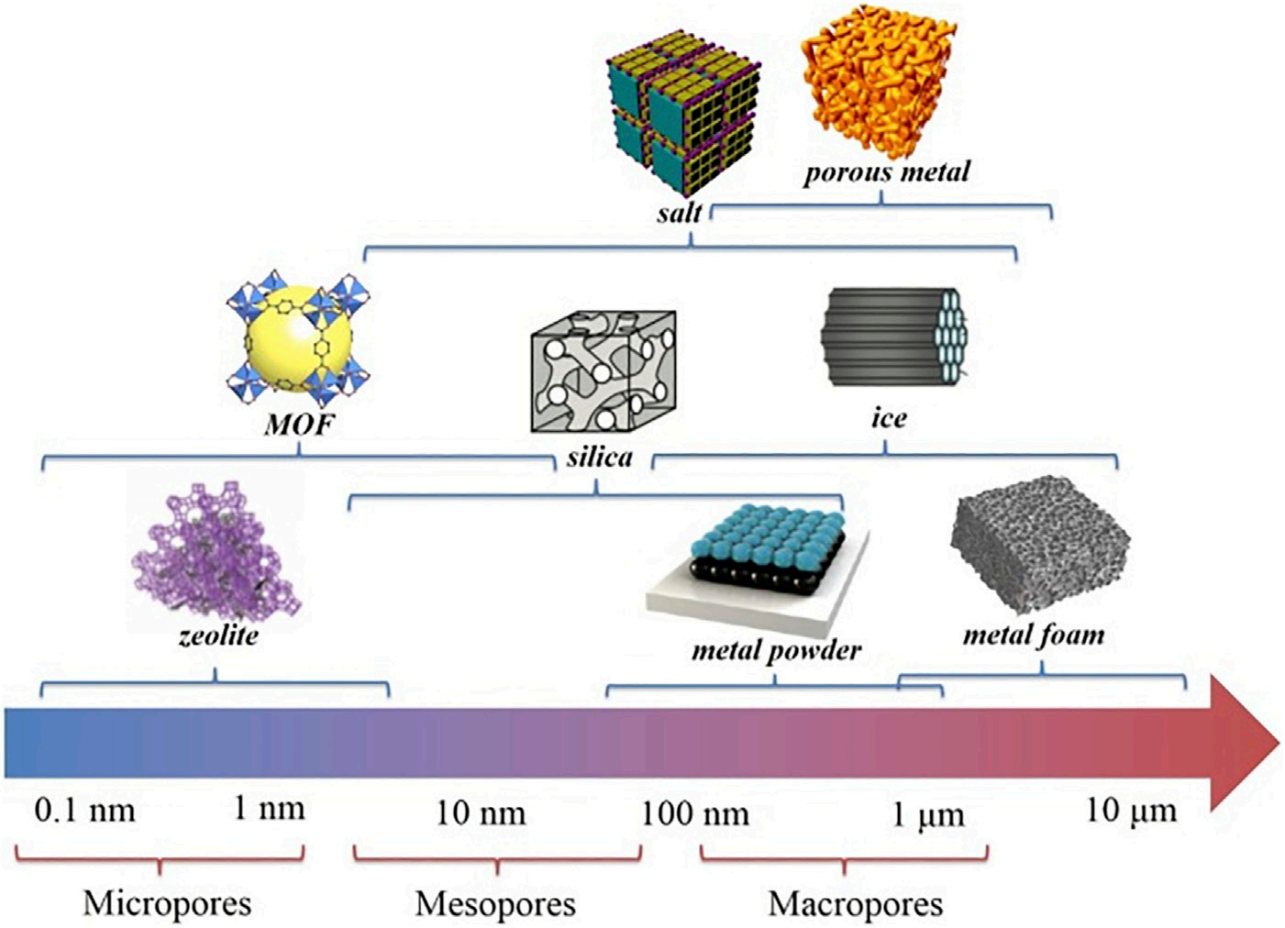
Different types of hard templates and their corresponding length scales (Zhu et al., 2019).

Compared to soft templates, hard templates have better domain limitation and higher stability, so they can achieve a strict regulation of material size and morphology. And different template agents can be selected according to the requirements of the target product to achieve precise regulation of different specific surface areas, pore sizes, morphology, and other characteristics. As shown in Figure 4, Zhu et al. (Zhu et al., 2019) classified various hard templates according to the pore sizes of different templates and corresponding composites. It can be seen that different pore sizes correspond to different template materials, therefore, based on the performance requirements of the target product, selecting the most suitable template material, which is one of the key factors for the successful synthesis of template materials. However, hard templates are difficult to be removed; the majority of these templates act as supports, which limits the application of hard template method to a certain extent (Liu D. et al., 2016).

In practice, hard template method has excellent results in the treatment of industrial wastewater, such as the adsorption of heavy metal ions. Currently, numerous types of hard templates have been investigated, such as silica (Fuertes et al., 2005), metal–organic frameworks (MOF) (Amali et al., 2014), mesoporous carbon (Libbrecht et al., 2015), poly (methyl methacrylate) (PMMA) (Liu et al., 2018), etc., and the specific materials are described below.

#### 2.1.1 Mesoporous Silica Templates

Mesoporous silica has a high specific surface area and good hydrophobicity. When used as a hard template for wastewater adsorption treatment, it can improve the morphology and pore structure of the material very well, and is commonly used as a hard template for the synthesis of mesoporous carbon materials in practical applications. Mesoporous silica has a three-dimensional interconnected pore structure, which offers the possibility of introducing carbon precursors. On the other hand, the three-dimensional interconnected pore structure of mesoporous silica also ensures the successful synthesis of mesoporous carbon, rather than obtaining carbon materials with nanowire or rod-like structures.

The preparation process usually starts by immersing the carbon precursor into the pore channel of the template agent to cause polymerization, and then removing the mesoporous silica template with hydrofluoric acid or sodium hydroxide solution after carbonization. The ordered mesoporous carbon CMK-1 was successfully prepared using mesoporous silicas MCM-48 as templates and sucrose as carbon precursors, heated to 1173 K and carbonized, and removed the silicon template using dissolution in sodium hydroxide solution. Moreover, they obtained samples Cn-CMK-1 by controlling the pore size of the template, and the specific surface area and pore size of the samples were 1700 m^2^/g, 1.24 cm^3^/g (C16-CMK-1), 1800 m^2^/g, 1.21 cm^3^/g (C18-CMK-1), and 1710 m^2^/g, 1.23 cm^3^/g (C20-CMK-1), respectively (Kruk et al., 2000). Similarly, Jun et al. (Jun et al., 2000) used ordered mesoporous silica SBA-15 as a template agent and sucrose as the carbon source, carbonized the mixture at 1173 K, and finally the ordered mesoporous carbon CMK-3 was obtained by removing the mesoporous silica template using sodium hydroxide or by washing with hydrofluoric acid. The specific surface area of CMK-3 was 1520 m^2^/g, and the total pore volume was 1.3 cm^3^/g.

Kim et al. (2005) used mesoporous silica SBA-16 as a template agent; and sucrose, furfuryl alcohol, and acenaphthene as carbon precursors to synthesize cubic mesoporous carbon, respectively. The reactions were all carbonized at 1173 K, and the mesoporous silica templates were removed with hydrofluoric acid. The experimental results showed that the furfuryl alcohol-based mesoporous carbon has a high specific surface area and total pore volume (2050 m^2^/g, 2.33 cm^3^/g), which is higher than that of sucrose-based mesoporous carbon (1370 m^2^/g, 1.20 cm^3^/g) and acenaphthene-based mesoporous carbon (1260 m^2^/g, 1.68 cm^3^/g). It further shows that the selection of suitable silicon templates and carbon precursors can effectively control the mesoporous structure of the materials.

#### 2.1.2 Carbon Templates

Carbon materials, such as mesoporous carbon and carbon nanotubes, are also used as template agents. Among them, mesoporous carbon is stable and has an ordered pore structure, so it is often used as a template agent to synthesize ordered mesoporous materials such as nanoscale zeolite molecular sieves and mesoporous metal oxides. The process of synthesizing mesoporous carbon into a mesoporous material usually involves replicating the pore channels of the mesoporous carbon into the new mesoporous material and removing the mesoporous carbon template under certain conditions. Roggenbuck et al. (2006) chose mesoporous carbon CMK-3 as a hard template, infiltrated the carbon matrix with Mg(NO_3_)_2_, removed the template by heating, and later obtained mesoporous MgO. The specific surface area and total pore volume of mesoporous MgO were 280 m^2^/g and 0.52 cm^3^/g.

Carbon nanotubes can greatly improve the reactivity of the material due to their characteristic pentagonal defect structure. Liu et al. (2015) prepared MWCNTs@carbon nanocables by a hydrothermal synthetic method using multi-walled carbon nanotubes (MWCNTs) as a template agent and glucose as a carbon source, and used anhydrous ethanol for washing to remove the template during the preparation.

#### 2.1.3 Metal–Organic Framework Polymer Templates

Metal–organic frameworks (MOFs) are a new class of materials with a three-dimensional pore structure, in which specific surface areas are significantly high (in the range of 1000–10000 m^2^/g), even higher than that of zeolites (3500 m^2^/g). Also, based on the inherent instability of the MOFs, it is easy to remove when they were used as template agents (Zhao et al., 2021).

ZIF-8, as one of the typical MOF materials, has excellent chemical and thermal stability and is an ideal template for the preparation of the material. The ZnS nanocages were prepared using ZIF-8 as template agents and thioacetamide as the sulfur precursor, and the ethanol solution was chosen to remove the template during the preparation process (Jiang et al., 2012). Zhang et al. (2018) prepared PDA/PEG@ZIF-8 composite nanoparticles by grafting polyethylene glycol (PEG) polymers by the template method using ZIF-8 as a hard template. The ZIF-8 template was further removed by etching with dilute hydrochloric acid adjusting the pH to 7 to obtain PDA/PEG composite nanocapsules.

#### 2.1.4 Other Hard Templates

Nanoporous anodic alumina, palygorskite (Pal) and spherical particles (PMMA microspheres, CaMg(CO_3_)_2_ microspheres) are also widely used as hard templates for wastewater adsorption. Mohajeri et al. (2017) used nanoporous anodic alumina as a template agent, ethylene as a carbon precursor, and argon as a carrier gas to synthesize ordered carbon nanotube arrays by chemical vapor deposition (CVD), and the template was removed by pyrolysis during the preparation. Zhong et al. (2019) used modified palygorskite (Pal) as a hard template and glucose as a carbon source to synthesize carbon-coated palygorskite (Pal@C) by impregnation and carbonization, with further final acid-base treatment to remove the Pal template to obtain amorphous carbon nanotubes (ACNT).

Spherical particles were also successfully applied as hard templates for the synthesis of adsorbents, such as poly (methyl methacrylate) (PMMA) microspheres. Using PMMA colloidal microspheres as template agents can synthesize three-dimensional hierarchical porous graphene-Fe_3_O_4_ (3D-HPGF) nanocomposites, and the PMMA templates were removed by calcination at 873 K under nitrogen atmosphere (Liu et al., 2018). Similarly, by using CaMg(CO_3_)_2_ microspheres as a template agent and Al(NO_3_)·9H_2_O as an aluminium source, Al_2_O_3_ hollow microspheres can be successfully synthesized with further calcinations, and the CaMg(CO_3_)_2_ microsphere template was also removed by calcination during the preparation process (Tian et al., 2016).

### 2.2 Soft Template

**FIGURE 5:**
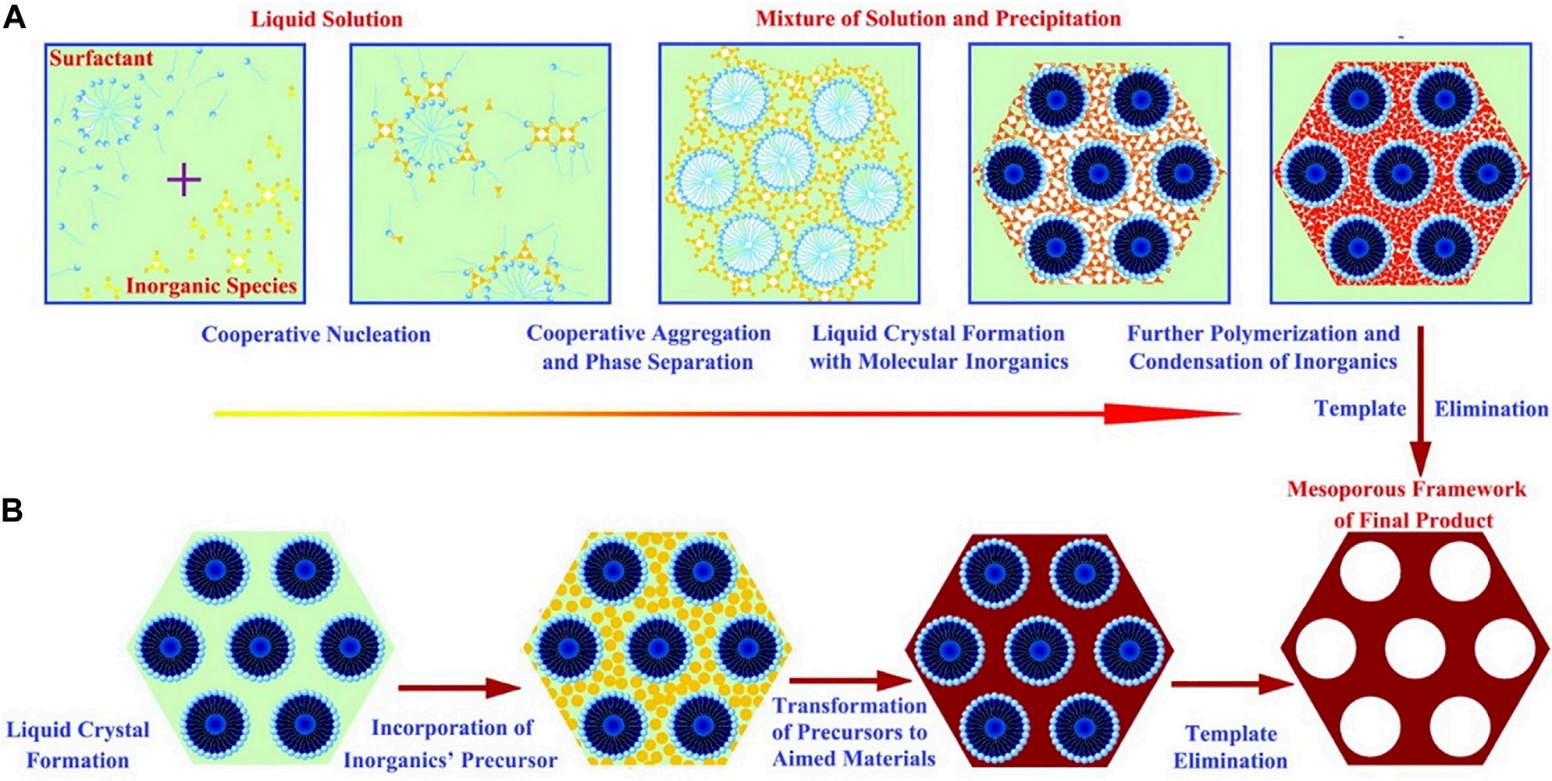
Mechanism of the soft template method (Wan and Zhao, 2007).

Soft templates mainly rely on intermolecular interaction forces to maintain the stability of the structure, and the material diffuses through the cavity walls into the interior of the static pore. Wan and Zhao (2007)schematically summarized the two mechanisms for the synthesis of soft templates (Figure 5), including the cooperative self-assembly and liquid-crystal templating processes. As shown in Figure 5A, cooperative self-assembly is based on inorganic–organic interactions between inorganic and surfactants. In this pathway, inorganic species and surfactants cooperate to nucleate as well as form liquid crystals with molecular inorganics, removing the template after further polymerization and condensation of inorganics. As shown in Figure 5B, the “true” liquid crystal templating process is that the liquid crystal mesophases are involved in the templating assembly to synthesize aimed materials, which means the liquid crystals are incorporated directly with precursors, and the template is removed after the transformation of the precursor to target product by reaction.

The advantages of the soft template method typically include simple construction, relatively mild experimental conditions, and its capacity to synthesize different materials with various morphologies. However, it has low structural stability compared to the hard template method, which results in it being a less efficient template. Typically, organic molecules are used as soft templates, such as micelles (Zhao et al., 2006; Gao et al., 2015), emulsified droplets (Bourret and Lennox, 2010; Wang B. et al., 2011; Pang M. et al., 2013), biological molecular assemblies (Yan et al., 2017; Zhao et al., 2017; Wang Y. et al., 2020), gas bubbles (Fan et al., 2004; Yan and Xue, 2007), etc., and the specific materials are described below.

#### 2.2.1 Micelle Templates

Micelles, refer to the aggregates by the mutual attraction of hydrophobic parts of surfactants after they are dissolved in water, which have excellent solubilization effects, are extremely common and highly versatile soft template materials. However, during the preparation process, a single aggregated micellar template tends to be very sensitive to the solvent, resulting in it being unstable in the reaction. In the recent years, it has been found that the introduction of surfactants in the preparation process can effectively improve this phenomenon. For example, Zhao et al. (2006) prepared SnO_2_ hollow spheres using micelles composed of terephthalic acid and sodium dodecylbenzene sulfonate (SDBS) as templates, and successfully constructed hollow structures for the materials. Gao et al. (2015) used worm-like micelles as soft templates to prepare Au and Au-based alloy nanowire networks with adjustable composition ratios. The worm-like micelles were obtained by mixing HAuCl_4_ and 3-(N,N-dimethylpalmitylammonio) propanesulfonate (PAPS) in an aqueous solution, and the AuAg and AuPt alloy nanowire networks were synthesized by a stepwise reduction method using NaBH_4_-PAPS and ascorbic acid (AA)-PAPS mixtures as reductants.

#### 2.2.2 Emulsion-Based Templates

Emulsified droplets, a non-homogeneous system formed within a mixture of two and more incompatible liquids, are an important class of soft templates. During the preparation process, emulsion droplets usually obtain materials with hollow structures through sol-gel coating, interfacial precipitation, and hydrothermal reactions. Pang M. et al. (2013) chose polyoxyethylene and sorbitan trioleate to form emulsified droplets, and used them as templates to synthesize Fe-soc-MOF cubes *via* solvothermal method. After removing the droplets, Fe-soc-MOF cubic hollow colloids with different sizes and arrangements with a specific surface area of 1100 m^2^/g, and a total pore volume of 0.49 cm^3^/g were obtained.

Oil-in-water (O/W) microemulsions and water-in-oil (W/O) microemulsions are two important types of emulsified droplets. Wang B. et al. (2011) used glycerol/water as emulsion templates and Fe_2_O_3_ nanoparticles as raw materials to synthesize α-Fe_2_O_3_ hollow spheres with lamellar substituents under hydrothermal conditions. The prepared templated material has a large cavity space and well-defined shell layer, and has a specific surface area of 103.3 m^2^/g and a wide pore size distribution. Similarly, the W/O microemulsion templates have been widely applied at present. For example, Bourret et al. (Bourret and Lennox, 2010) used CH_2_Cl_2_ solution as the aqueous phase of the microemulsion and obtained copper hydroxide nanofibers (NF) by interfacial reaction in a two-phase system (H_2_O/CH_2_Cl_2_). As the reaction proceeds, porous microspheres with shell-like structures were finally synthesized, and the prepared CuS nanohollow spheres have a diameter of about 200 nm and a wall thickness of about 30 nm.

#### 2.2.3 Biological Templates

Biological templates, such as biopolymers, cellular tissues, bacteria, and DNA, are some of the most common soft templates, because of their wide source, non-toxicity, non-contamination, and low cost, they have a great potential in the development of template agents. Zhao et al. (2017) treated rape pollen grains with anhydrous ethanol and 8 M hydrochloric acid, respectively, and dried at room temperature to obtain two types of pollen templates. These two pollen templates were used to synthesize ZrO_2_ hollow microspheres with scale-like outer walls (S-1) and porous hollow ZrO_2_ microspheres (S-2) via the same experimental method, that is, treated by microwave solvothermal method at 110°C, washed with distilled water and absolute ethanol, dried, and calcined at 580°C. The specific surface areas of S-1 and S-2 are 27.99°m^2^/g and 40.92°m^2^/g, respectively, while the average pore diameters are 14.57 and 10.19°nm, respectively.

In addition, cotton is one of the most common biological templates. The dried cotton templates were dipped into the solution for hydrolysis treatment, followed by the reaction in the reactor under nitrogen atmosphere and washed with anhydrous ethanol and deionized water, and dried to obtain the corresponding cotton templated materials. For example, Yan et al. (2017) prepared fibrous magnesium oxide by template-directed synthesis using cotton as a template agent and hydrolyzed in cetyltrimethylammonium bromide solution. Wang Y. et al. (2020) used cotton as a template agent and hydrolyzed it in sulfuric acid solution to synthesize mesoporous carbon lanthanum-doped films (MC-La).

#### 2.2.4 Other Soft Templates

In addition to the template materials mentioned above, bubbles, vesicles, and amphiphilic polymers are also excellent materials for soft templates. The preparation process of bubble templates in the liquid phase usually consists of the following two steps: preparation of the template (generation of bubble emulsion), deposition/adsorption of particles on the template surface, and further growth/aggregation of particles (Fan et al., 2004). In addition, Yan and Xue (2007) used the CO_2_ bubbles generated during the reaction as a template agent to obtain ZnO hollow microspheres by pyrolysis.

Vesicles, as one of the most important microreactors for controlled growth, are important materials for soft templates. Zheng et al. (2002) obtained anionic sodium dodecyl sulfate (SDS) vesicles by ultrasonic irradiation and used them as soft templates. The cadmium ions were adsorbed on the surface of the templates by electrostatic interaction, and after washing with distilled water and anhydrous ethanol to remove the templates, CdSe hollow nanoparticles with the size of 100–200 nm were obtained.

Amphiphilic polymers can also be used as soft templates to synthesize hollow structural materials, and different morphologies of materials can be obtained by adjusting the size and composition of the template. For example, Lin et al. (Pang X. et al., 2013) used a star-shaped PS-b-PAA-b-PS triblock copolymer as soft templates to synthesize hollow gold nanoparticles.

## 3 Moderating the Behavior of Materials by the Template Method

### 3.1 Pore Structure Tuning

The size and nature of different template agents vary, and their introduction into the same material or different materials will result in different specific surface areas, pore sizes, and pore volumes; the pore sizes and volumes will show a certain variation with the concentration of the template agent. In general, smaller sized and more concentrated template agents are more favorable for the preparation of templated materials.

#### 3.1.1 Effect of Template Agent Type on Pore Structure

The specific surface area, pore volume, and pore size of the synthesized materials varied considerably depending on the template agent (Table 1). The pore size of the materials depends on the size of the template agent used. When the radius of the template agent is smaller than the pore size of the precursor, it can be easily incorporated into the precursor, thus limiting the pore shrinkage of the material during the reaction process. After the reaction, when the radius of the template agent is still smaller than the pore size of the synthetic material, the pore structure will remain after the template agent is removed, resulting in larger pore size of the material and further increasing its specific surface area and pore volume. Therefore, the template with a smaller pore diameter than the material should be selected.

**TABLE 1:** Structural parameters of the samples synthesized with different templates.

Sample	Template agent	Specific surface area (m^2^/g)	Pore volume (cm^3^/g)	Average pore size (nm)	References
Al_2_O_3_ (CTAB)	Cetyltrimethylammonium bromide (CATB)	335.7	0.66	6.1	Bo and Jing (2007)
Al_2_O_3_ (CA)	Sodium laurate (CA)	312.0	0.44	4.1	
Al_2_O_3_ (P123)	PPO-PEO-PPO (P123)	318.8	1.00	9.8	
Aluminosilicate materials (CTAB)	Cetyltrimethylammonium bromide (CTAB)	211.5	0.31	4.4	Ma et al. (2020)
Aluminosilicate materials (DETA)	Diethylenetriamine (DETA)	75.2	0.12	4.8	
Aluminosilicate materials (CMC)	Sodium carboxymethylcellulose (CMC)	161.5	0.37	6.2	
Aluminosilicate materials (SDS)	Sodium dodecyl sulfate (SDS)	144.3	0.27	5.4	
Aluminosilicate materials (F68)	PEO_80_-PPO_30_-PEO_80_ (F68)	277.6	0.24	6.0	
Aluminosilicate materials (F108)	PEO_133_-PPO_50_-PEO_133_ (F108)	289.7	0.24	6.5	
S7	F127 (EO_106_PO_70_EO_106_)	1022	1.35	5.3	Yang et al. (2006)
S14	F127 + PEG	1145	0.82	2.9	
Mesoporous Titania (TS-0.1)	Cetyltrimethylammonium chloride (CTAC)	83.8	0.17	9.0	Yusuf et al. (2003)
Mesoporous Titania (TP-0.1)	Polyethylene glycol (PEG)	57.1	0.094	8.4	
Mesoporous Titania (TPS-0.01)	CTAC + PEG	39.6	0.076	6.2	
Nano ferric oxides (C-Fe_2_O_3_)	Cetyltrimethylammonium bromide (CTAB)	24.8	0.11	11.9	Lei et al. (2018)
Nano ferric oxides (G-Fe_2_O_3_)	Glycerine (GI)	23.6	0.091	10.2	
Nano ferric oxides (T-Fe_2_O_3_)	Tartaric acid (TA)	40.8	0.092	4.8	
ZSM-5 Zeolite (NZ)	Tetrapropylammonium hydroxide (TPAOH)	364.0	0.39	—	Zhang et al. (2015)
ZSM-5 Zeolite (MZ)	Cetyltrimethylammonium bromide (CTAB)	374.0	0.45	—	
ZSM-5 Zeolite (NSZ)	N-octadecyl-N′-hexyl-tetramethyl-1,6-hexanediaminium (C_18-6-6_Br_2_)	505.0	0.63	—	

Using different sizes of template agents will produce materials with different structures. Some synthetic materials have many mesopores, as well as slit pores formed by the accumulation of particles, which all can lead to an uneven distribution of pore sizes in the synthesized material and thus affect the structural parameters of the material. Therefore, the specific surface area, pore diameter, and pore volume of the same material synthesized with different templates are different. For instance, mesoporous alumina was prepared using cetyltrimethylammonium bromide (CATB), sodium laurate (CA), and poly (propylene oxide)-Poly (ethylene oxide)-Poly (propylene oxide) (PPO-PEO-PPO, P123) as template agents, respectively (Bo and Jing, 2007); aluminosilicate mesoporous materials were synthesized using cetyltrimethylammonium bromide (CTAB), diethylenetriamine (DETA), sodium carboxymethylcellulose (CMC), sodium dodecyl sulfate (SDS), and polyethylene glycol-polypropylene glycol-polyethylene glycol (PEO_80_-PPO_30_-PEO_80_, F68 and PEO_133_-PPO_50_-PEO_133_, F108) as template agents correspondingly (Ma et al., 2020); and silica spheres were prepared using pluronic triblock copolymer (EO_106_PO_70_EO_106_, F127), F127, and PEG respectively as template agents (Yang et al., 2006).

#### 3.1.2 Effect of Template Agent Concentration on Pore Structure

The concentration of the template agent also has a large effect on the specific surface area, pore volume, and pore size of the synthetic material. As the concentration of the template agent increases, the average pore size of the material gradually increases, and the pore capacity also increases accordingly. Nevertheless, the change in specific surface area is related to the porosity of the synthetic material, so for different synthetic materials, the specific surface area has a different change pattern of the change in mass concentration.

For example, the use of polyethylene glycol (PEG) and/or cetyltrimethylammonium chloride (CTAC) as templates enables the preparation of mesoporous titanium dioxide (Table 2). It was shown that with the increase of CTAC and PEG concentration, the specific surface area, average pore size, and pore volume of the gel increased. These structural parameters of gels prepared from CTAC solution (TS-0.1), gels prepared from PEG solution (TP-0.1), and dry gels prepared from CTAC and PEG were higher than those without additives (TiO_2_-0), and the specific surface area and pore size of TS-0.1 were even about twice as large than those of TiO_2_-0 (Yusuf et al., 2003). The average pore size and pore volume of the silica-aluminate mesoporous material synthesized using F108 as a template agent gradually increased with increasing mass concentration of F108, but the specific surface area gradually decreased (Ma et al., 2020).

**TABLE 2:** Structural parameters of the samples synthesized with different concentrations of templates.

Template agent	Concentrations (g/ml)	Specific surface area (m^2^/g)	Pore volume (cm^3^/g)	Average pore size (nm)	References
PEO_133_-PPO_50_-PEO_133_ (F108)	0.01	297.6	0.23	6.0	Ma et al. (2020)
	0.02	269.8	0.25	6.4	
	0.03	237.6	0.26	7.2	
	0.04	204.6	0.27	11.7	
Cetyltrimethylammonium chloride (CATC)	0	38.6	0.063	5.2	Yusuf et al. (2003)
	0.001	54.2	0.078	4.6	
	0.01	56.6	0.088	5.0	
	0.05	72.8	0.13	7.6	
	0.1	83.8	0.17	9.0	
Polyethylene glycol (PEG)	0.01	27.8	0.041	4.8
	0.05	47.3	0.067	4.6
	0.1	57.1	0.094	8.4

### 3.2 Morphology Tuning

The introduction of template will change the microstructure of materials. Different template agents will get different structures, such as porous structure, worm-like structure, core-shell structure, etc. As the mass transfer of contaminants within an adsorbent is largely dependent on the pore structure of the material, this section will focus on porous materials, which are subdivided into disordered and ordered pore structures.

#### 3.2.1 Disordered Porous Structure

**FIGURE 6:**
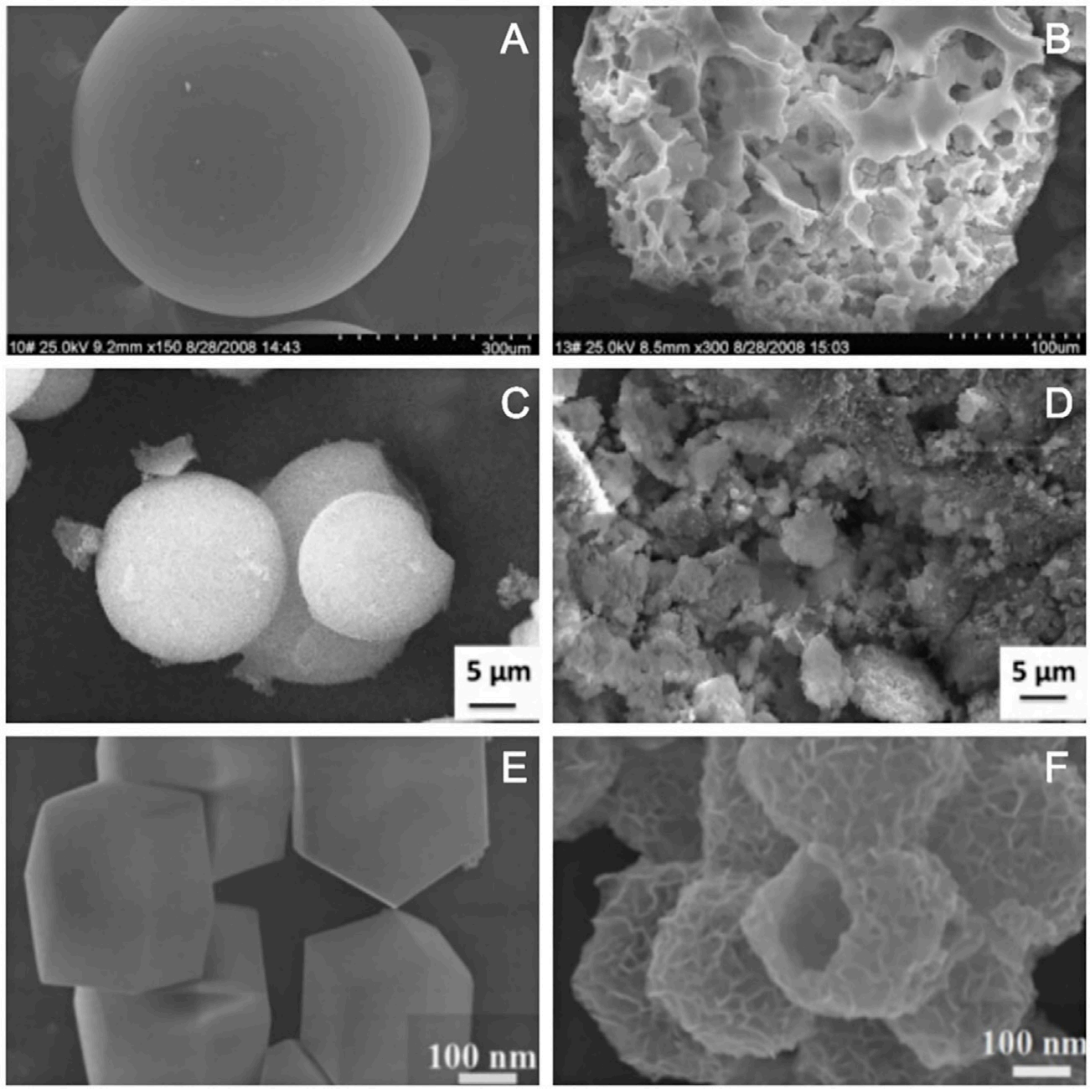
SEM images of D311 resin **(A)** and PCTOM **(B)** (Zhang et al., 2011); SiO_2_ **(C)** and Fe_2_O_3_/SiO_2_ composite monoliths **(D)** (Singh et al., 2018); ZIF-67 **(E)** and NiCo-LDH **(F)** (Hu et al., 2019).

The template method is mainly used to make the porous structure on the surface of materials or to make the surface rough. In some cases, the porous structure is disordered. Considering the template to be the skeleton, the material with pore structure can be obtained by solution etching, for example, using D311 resin as the template skeleton to synthesize porous nano-calcium titanate microspheres (PCTOM). The D311 resin is a sphere with a smooth surface (Figure 6A); the surface of the synthesized material became rough and disordered porous structure was obtained (Figure 6B; Zhang et al., 2011). SiO_2_ is a spherical particle with smooth surface (Figure 6C). Considering it to be the template skeleton and introducing the precursor Fe_2_O_3_, the Fe_2_O_3_/SiO_2_ composite monoliths with rough surface and pore structure can be obtained (Figure 6D; Singh et al., 2018). Zeolitic imidazolate framework-67 (ZIF-67) is a dodecahedral structure with a smooth surface (Figure 6E), which is used as a template skeleton to synthesize hierarchical porous Ni/Co-layered double hydroxide (NiCo-LDH), and the NiCo-LDH is a hollow dodecahedron with porous structure (Figure 6F; Hu et al., 2019).

**FIGURE 7:**
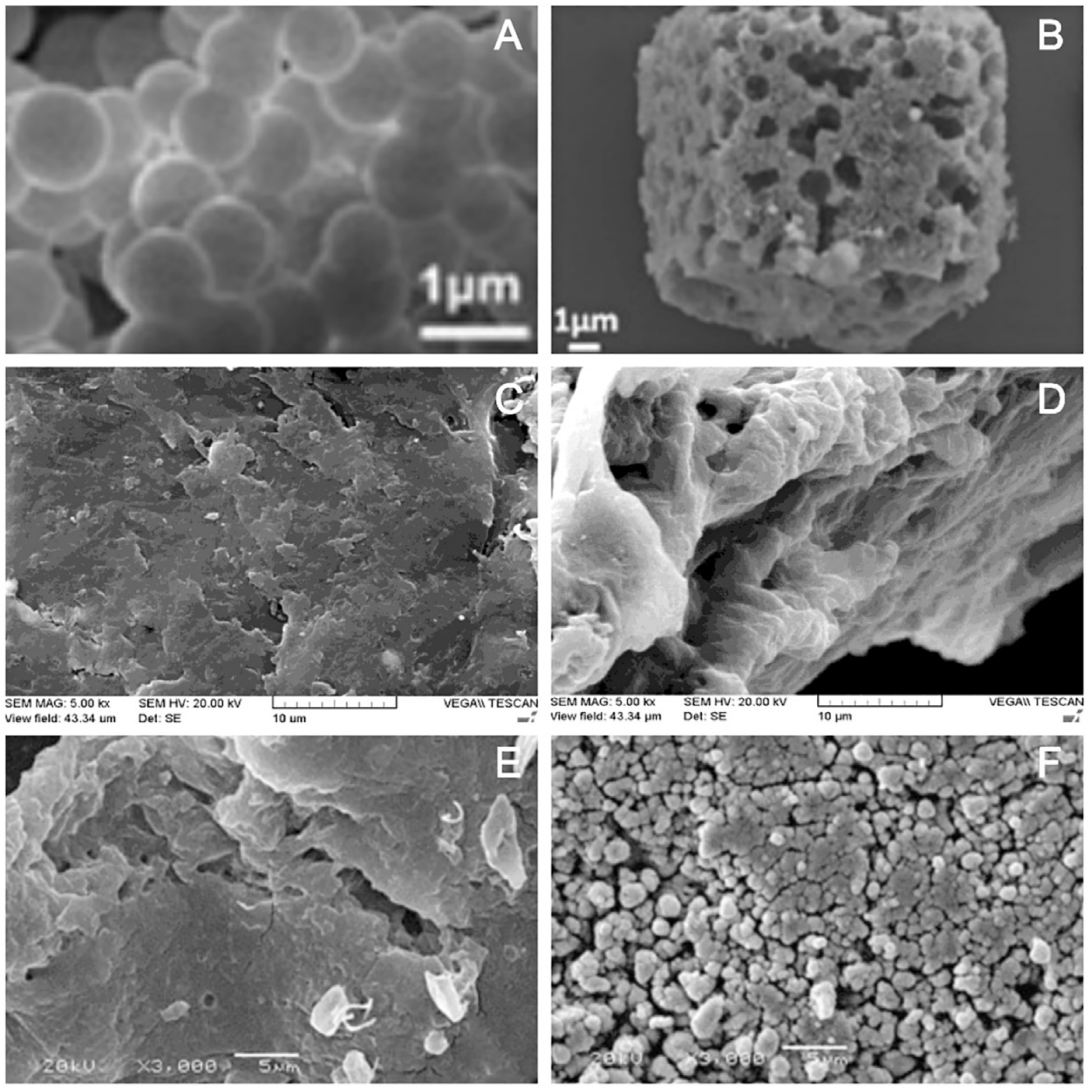
SEM images of carbon sphere **(A)** and DP Mn_2_O_3_-carbon-PVP cubes **(B)** (Shao et al., 2017); CTS **(C)** and TCCTS **(D)** (Dai et al., 2012); NSC **(E)** and crosslinked NSC resin **(F)** (Sun et al., 2007).

The disordered porous material with rough surface can also be obtained by using porous material as the precursor, meanwhile, taking it as skeleton and introducing template into its pores. The carbon spheres are smooth spheres (Figure 7A) and when used as templates for the synthesis of double porous Mn_2_O_3_ (DP Mn_2_O_3_-carbon-PVP) cube, the carbon spheres embedded the precursor well and the final material with rough surface and porous structure was obtained (Figure 7B; Shao et al., 2017). The surface of the chitosan (CTS) is relatively smooth and the structure is compact (Figure 7C). After introducing the template Fe(III), the surface of the thiourea cross-linked chitosan (TCCTS) obtained is rough and the structure is incompact and reticular with disordered pore structure (Figure 7D; Dai et al., 2012). Crosslinked NSC resin was synthesized with N-succinyl-chitosan (NSC) as the precursor skeleton and Cu (II) as a template agent. After the introduction of template, the surface structure of crosslinked NSC resin was denser than that of NSC and has a porous structure (Figures 7E,F; Sun et al., 2007).

#### 3.2.2 Ordered Porous Structure

**FIGURE 8:**
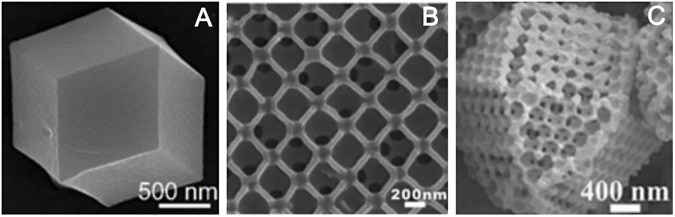
SEM images of ZIF-8 **(A)** (Salunkhe et al., 2016); HKUST-1 **(B)** (Wu et al., 2011); and SOM-ZIF-8**(C)** (Shen et al., 2018).

**FIGURE 9:**
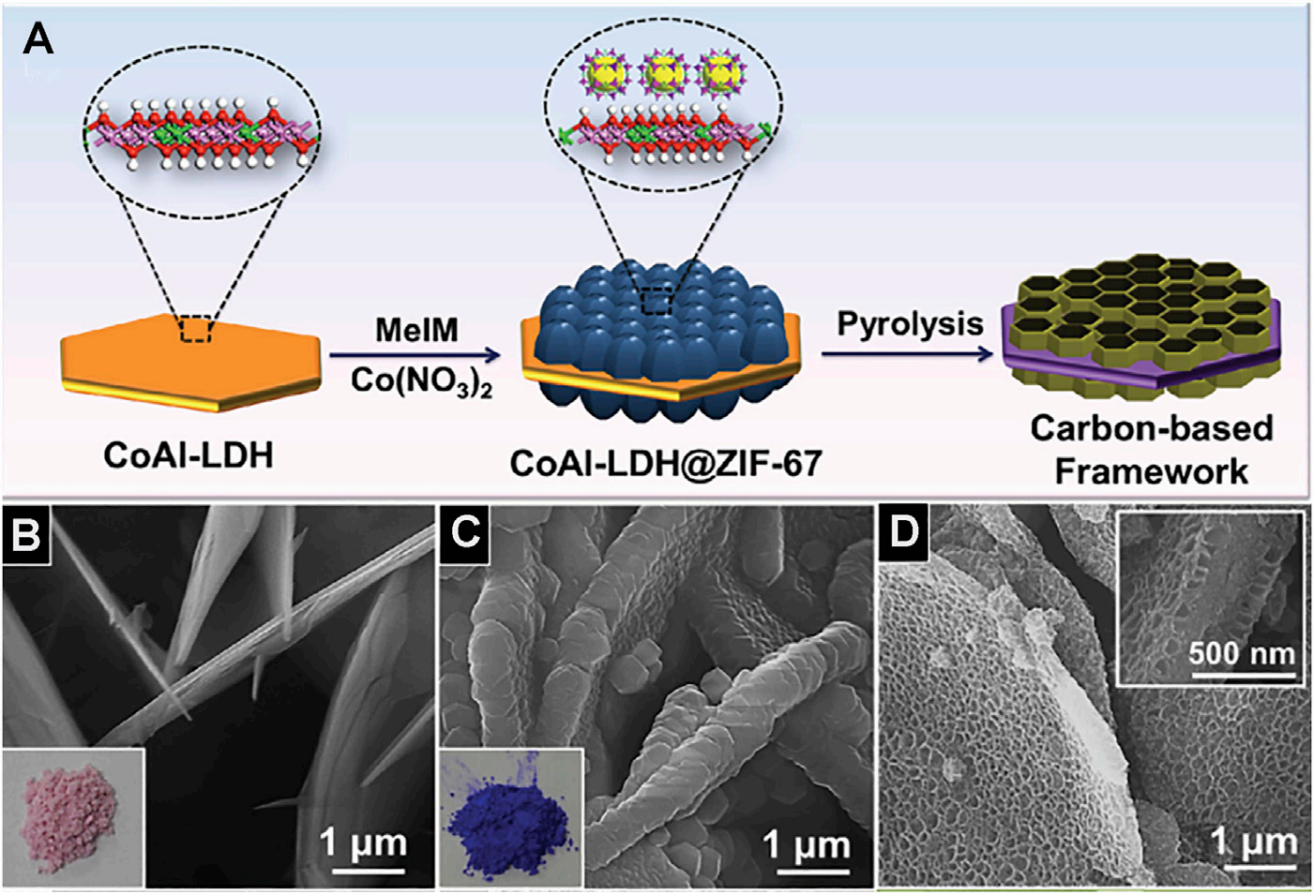
**(A)** Schematic illustration for the synthesis of porous carbon-based framework. SEM images of CoAl-LDH **(B)**, CoAl-LDH@ZIF-67 **(C),** and LDH@ZIF-67-800 **(D)** (Li et al., 2016).

Template method can also construct the ordered porous structure. Metal–organic framework polymer is one of the most typical examples, although different templates will get different structural materials, they almost have ordered pore structure. For instance, metal–organic frameworks (MOFs) ZIF-8 synthesized by the template-free method has a dodecahedral shape, but without porous structure (Figure 8A; Salunkhe et al., 2016). Using polystyrene (PS) as a template agent, MOFs precursor solution was introduced to infiltrate the template. After removing the template, HKUST-1 crystal was obtained. HKUST-1 has three-dimensionally ordered and interconnected macropores (Figure 8B; Wu et al., 2011). PS has adjustable and stable morphology in methanol solution (MeOH). After the precursor infiltrates the template PS, it exposed the precursor@PS to NH_3_/MeOH solution and SOM-ZIF-8 with three-dimensionally ordered macropores can be obtained (Figure 8C; Shen et al., 2018). Using CoAl-layered double hydroxide (CoAl-LDH) as a template skeleton, and introducing ZIF-67 to synthesize CoAl-LDH@ZIF-67, the final material LDH@ZIF-67-800 was obtained after the removal of the template by pyrolysis and the synthetic path is shown in Figure 9A. LDH@ZIF-67-800 is a surface ordered porous structure (Figure 9C; Li et al., 2016).

**FIGURE 10:**
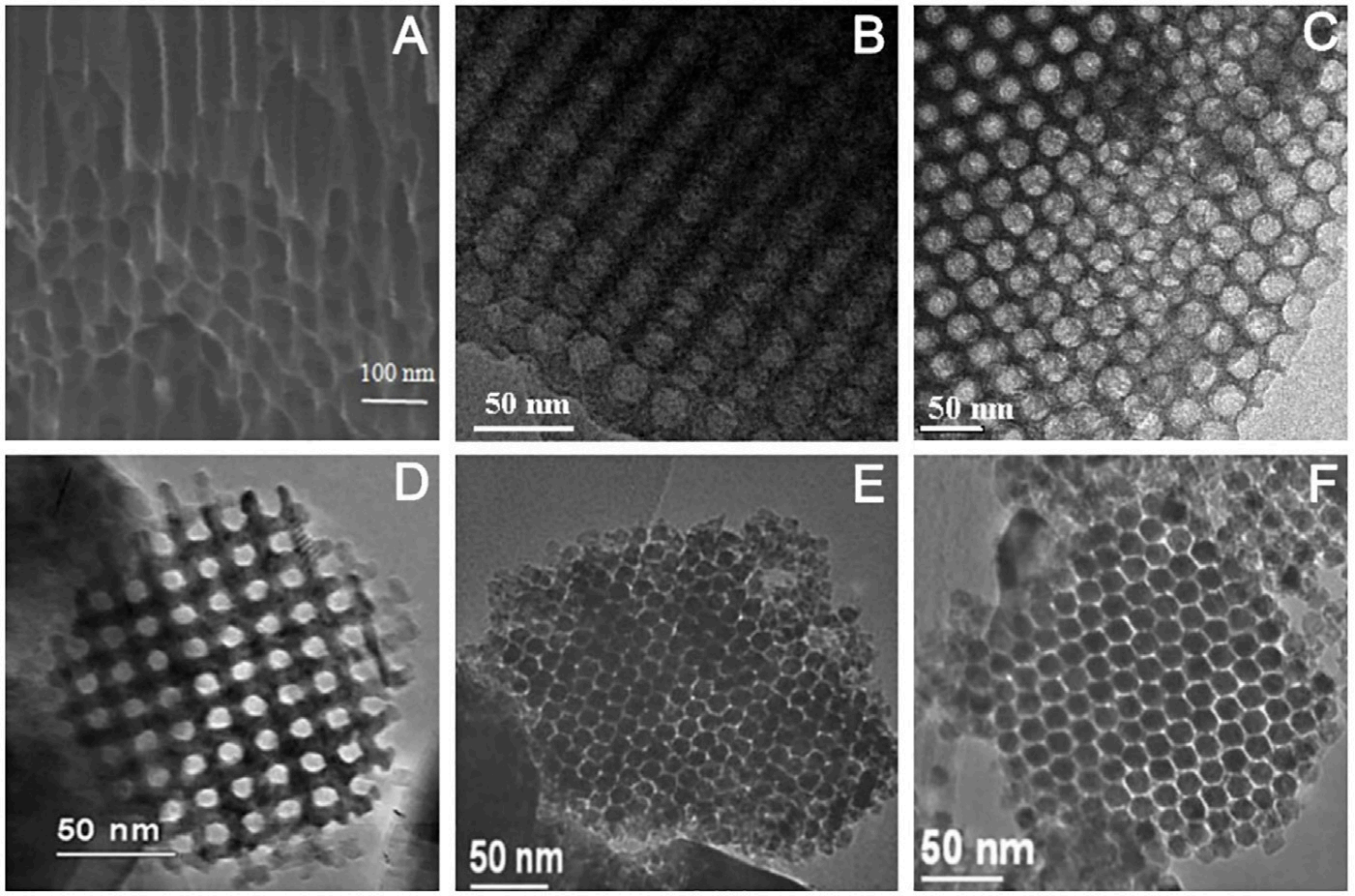
SEM images of s of CNTs arrays/NPAA composite membrane **(A)** (Mohajeri et al., 2017); TEM images of C-FDU-18-800 **(B)** and Si-FDU-18-HC **(C)** (Deng et al., 2007); Cr_2_O_3_ **(D),** (Jiao et al., 2005), In_2_O_3_ **(E),** and Co_3_O_4_ **(F)** (Yue and Zhou, 2007).

In addition to MOF materials, ordered porous structures can also be obtained for some other templated materials. For example, using nanoporous anodic alumina (NPAA) as the template skeleton, the synthesized carbon nanotube arrays have a highly ordered pore structure (Mohajeri et al., 2017; Figure 10A). The mesoporous carbon (C-FDU-18-800) and mesoporous silica (Si-FDU-18-HC), synthesized using poly (ethylene oxide)-b-polystyrene (PEO-b-PS) diblock copolymers as templates, have highly ordered mesoporous structures (Figures 10B,C; Deng et al., 2007). Similarly, metal oxides such as Cr_2_O_3_ (Figure 10D; Jiao et al., 2005), In_2_O_3_ (Figure 10E), and Co_3_O_4_ (Figure 10F; Yue and Zhou, 2007), synthesized with mesoporous silica as the template skeleton, all have regular mesoporous structures.

## 4 Application to Typical Pollutants in Wastewater

Current research on templated materials as adsorbents is focused on two types of pollutants, namely dyes and heavy metals. As mentioned above, the template method can effectively modulate the morphological and structural parameters such as pore size, pore volume, and specific surface area of materials, which are the direct factors affecting the adsorption performance. In addition to enormous specific surface area and rich pore structure, the introduction of templates to modulate the surface functional groups and charges can achieve significant improvements in the adsorption performance of the target pollutants.

### 4.1 Heavy Metals

Heavy metals in water, even at very low concentrations, are extremely harmful to humans and the ecology. Currently, templated materials have been widely used for the adsorption of heavy metal ions in water like Ni(II), Co(II), Cu(II), Cr(III), Pb(II), etc., and have exhibited relatively outstanding adsorption capacities (Table 3). For example, using attapulgite (ATP) as a template agent, a novel polyaniline/attapulgite (PANI/ATP) composite material can be obtained by oxidative polymerization. The PANI/ATP exhibited an excellent adsorption capacity for Hg(II) (824 mg/g) (Cui et al., 2012), which was much higher than those of PANI (600 mg/g) (Wang et al., 2009)– amine resins (156–556 mg/g) (Zhu and Alexandratos, 2005; Alexandratos, 2007), aniline-m-sulfophenylenediamine copolymer (PANSP) (498 mg/g) (Lü et al., 2007), chitosan beads (323 mg/g) (Li et al., 2005), and mercaptopropyl mesoporous adsorbent (461 mg/g) (Brown et al., 2000). Using SiO_2_ monoliths as a template agent to synthesize metal oxide monoliths (Fe_2_O_3_/SiO_2_), the Fe_2_O_3_/SiO_2_ demonstrated high adsorption capacities for Pb(II) (850 mg/g) and Cr(III) (770 mg/g) (Singh et al., 2018), which were much higher than those of Fe_2_O_3_ monoliths (166 mg/g) (Singh et al., 2018), carbon coated monoliths (72 mg/g) (Teoh et al., 2013), and mesoporous silica (184 mg/g) (Awual and Hasan, 2014) for Pb(II), and those of Fe_2_O_3_ monoliths (125 mg/g) (Singh et al., 2018), modified lignin (68 mg/g) (Demirbaş, 2005), and borax sludge (16 mg/g) (Senberber et al., 2017) for Cr(III).

**TABLE 3:** Properties of various template materials and their ability to adsorb heavy metals.

Samples	Template agent	Surface area (m^2^/g)	Adsorbate	Adsorption capacity (mg/g)	Equilibrium time (min)	pH	Kinetics	Thermodynamics	Isotherms	Cycle numbers	References
C_KIT-6_	KIT-6	821	Ni(II)	145	—	5	Pseudo second order	Spontaneous and endothermic	Langmuir	—	Marciniak et al. (2019)
			Co(II)	148	—						
Fe_2_O_3_/SiO_2_	SiO_2_	562	Cd(II)	690	120	6	Pseudo second order	Spontaneous and exothermic	Freundlich and Langmuir	—	Singh et al. (2018)
			Cr(III)	770							
			Pb(II)	850							
CF-S-N	SiO_2_	—	Cu(II)	197	200	7	Pseudo second order	Spontaneous and endothermic	Langmuir	—	Ren et al. (2017)
			Mn(II)	195							
			Cd(II)	187							
			Pb(II)	183							
PANI/ATP	ATP	95	Hg(II)	824	200	6	Pseudo second order	Spontaneous and endothermic	Langmuir	5	Cui et al. (2012)
MCMs	SiO_2_	682	U(VI)	294	120	4	Pseudo second order	Spontaneous and endothermic	Langmuir	5	Zhang et al. (2017)
NSHS	CaCO_3_	—	Pb(II)	267	—	—	Pseudo second order	Spontaneous and endothermic	Langmuir	—	Manyangadze et al. (2020)
MP-Silica	Citric acid	504	Ag(I)	115	30	1–7	Pseudo second order	—	Langmuir	—	Pongkitdachoti and Unob (2018)
C_ST_	F127	426	Ni(II)	126	—	5	Pseudo second order	Spontaneous and endothermic	Langmuir	—	Marciniak et al. (2019)
			Co(II)	135	—						
PCTOM	D311 resin	—	Pb(II)	30	142	6	Pseudo second order	Spontaneous and endothermic	Langmuir	—	Zhang et al. (2011)
			Cd(II)	30	18						
			Zn(II)	15	24						
TP-CMCBs	CaCO_3_	53	Cu(II)	342	30	6	Pseudo second order	—	Langmuir	5	Liu et al. (2021)
			Mn(II)	262	30						
			Ni(II)	243	30						
MSC	CTMAB-MA	239	Ni(II)	278	80	5–7.5	Pseudo second order	Spontaneous and endothermic	Langmuir	2	Liu et al. (2020)
			Cu(II)	390	30						
			Zn(II)	402	80						
			Pb(II)	452	40						
			Mn(II)	201	110						
			Cd(II)	438	60						
TCCTS	Fe(III)	—	Fe(II)	48	60	5	Pseudo second order	—	Langmuir	—	Dai et al. (2012)
			Fe(III)	72							
NiCo-LDH	Ni(NO_3_)_2_	267	Cr(VI)	100	50	—	Pseudo second order	—	Langmuir	5	Hu et al. (2019)
MSNs	CTAB	675	Fe(III)	21	50	—	Pseudo second order	—	Langmuir	4	Meng et al. (2018)

#### 4.1.1 Factors Affecting the Adsorption of Heavy Metals by Templated Materials

Like conventional adsorbents, adsorbents prepared by the template method are also mainly influenced by factors such as pH and temperature.

Solution pH is one of the most critical factors affecting the adsorption of heavy metal ions by templated materials. Normally, as the pH increases, the proton concentration decreases and the adsorption of heavy metal ions increases accordingly. Nevertheless, when the solution is in an alkaline environment, hydroxyl ions form precipitates of hydroxides with metal ions, resulting in a decrease in the adsorption capacity of the adsorbent. For example, as the pH value of the solution is increased from 1 to 5, the adsorption capacity of both Fe(II) and Fe(III) by the thiourea crosslinked-chitosan (TCCTS) synthesized with iron (III) as a template agent gradually increased, and thereby reaching a maximum at pH = 5. In the higher pH range, the adsorption capacity of TCCTS decreases sharply (Dai et al., 2012).

Temperature is also one of the important factors affecting the adsorption of heavy metal ions in solution, as it mainly affects mobility and chain flexibility of metal ions, resulting in an increase in the adsorption capacity of the adsorbent with increasing temperature. For example, as temperature was increased from 25 to 55°C, the adsorption capacity of Cu(II) by the templated material CF-S-N increased from 196 mg/g to 199 mg/g. The adsorption of Cu(II) by polyacrylamide/functionalized multi-walled carbon nanotube PAAm/FMWCNTs) composites (Abo-Zahra et al., 2021) and the adsorption of Ni(II) and Co(II) by ordered mesoporous carbon (Marciniak et al., 2019) show this trend.

Furthermore, the adsorption kinetics of the adsorbent synthesized by template method fit the pseudo-second-order kinetic model, indicating that chemisorption primarily promotes the adsorption of heavy metal ions. The relevant thermodynamic parameters were calculated to confirm that the adsorption process is spontaneous and endothermic for most of the adsorbents. And the adsorbents all fit more closely to the Langmuir adsorption model, therefore the adsorption of heavy metal ions onto the synthesized adsorbents can be considered as a monolayer adsorption process.

#### 4.1.2 Mechanisms of Heavy Metal Adsorption

As mentioned in the previous section, the introduction of a template agent can effectively regulate the specific surface area, pore diameter, and pore volume of materials and change the microscopic morphology of materials, all of which provide the possibility to improve the adsorption performance of the material. On the one hand, the pore structure constructed by the template method can provide wider diffusion channels and lower mass transfer resistance, which facilitates the rapid diffusion of heavy metal ions in the internal structure of the adsorbent. On the other hand, the large specific surface area and pore size distribution formed by the template method provide more active sites for the adsorbent. For instance, mesoporous carbon (MCMs), synthesized using ordered mesoporous silica as a template agent, has a specific surface area of 682 m^2^/g (Zhang et al., 2017), which is higher than other carbon-based nanomaterials such as graphene oxide supported polyaniline (141 m^2^/g) (Sun et al., 2013) and carbonaceous nanofibers (264 m^2^/g) (Sun et al., 2016). Correspondingly, MCMs have a good adsorption property for U(VI) of 294 mg/g, which is also higher than that of graphene oxide-supported polyaniline (245 mg/g) and carbonaceous nanofibers (125 mg/g) (Table 3).

For templated materials, in addition to the pore adsorption mechanism, ion exchange, electrostatic interaction, and surface complexation are also the predominant adsorption mechanisms of heavy metals. The introduction of templates can alter the surface chemistry of the materials, providing favorable ions and functional groups for adsorption, as well as altering the surface charge properties of the material, all of which contribute to the adsorption performance of the material. For instance, templated material mesoporous carbons (MCMs) containing a large number of oxygenated functional groups such as -OH and -COOH have good adsorption properties for cationic heavy metals such as U(VI) (Zhang et al., 2017). For materials with an intrinsic surface charge that is less conducive for the adsorption of ions, for example, natural attapulgite (ATP), which has negatively charged functional groups on its surface and does not have electrophoretic mobility equal to zero, was used as a template agent to polymerize aniline, the obtained polyaniline/attapulgite (PANI/ATP) exhibits a point of zero charge and achieved a maximum adsorption capacity of Hg(II) over 800 mg/g (Cui et al., 2012). After the introduction of the template, the positively charged hierarchical porous Ni/Co-layered double hydroxide (NiCo-LDH) hollow dodecahedra exhibiting excellent adsorption properties for the negatively charged Cr(VI) ions in aqueous solution, and the NO- 3 ions which initially on the surface of the NiCo-LDH were replaced by Cr(VI) ions after adsorption (Hu et al., 2019; Table 3).

### 4.2 Dyes

Wastewater discharged from the printing, electroplating, textile, and leather industries contains large amounts of toxic and non-biodegradable dyes that have an irreversible effect on humans and the environment. The use of templated materials for the adsorption of dyes such as bisphenol A, rhodamine B, and methylene blue in wastewater exhibit better adsorption properties than the most conventional adsorbents. For example, Al_2_O_3_ hollow microspheres (AHS) adsorbed as much as 690 mg/g of Congo Red (Tian et al., 2016). Its adsorption performance outperformed many other metal oxide adsorbents such as Co_3_O_4_-Fe_3_O_4_ hollow spheres (123 mg/g) (Wang X. et al., 2011), NiO nanospheres (440 mg/g) (Zhu et al., 2012), and MgO-MgFe_2_O_4_ composite (498 mg/g) (Han et al., 2014). The new hollow polydopamine microcapsules (H-PDA-MCs) showed a capacity of 179 mg/g for methylene blue (Feng et al., 2021), exceeding that of PDA microspheres (91 mg/g) (Fu et al., 2015), palm kernel fibres (95 mg/g) (El-Sayed, 2011), and magnetic γ- Fe_2_O_3_/SiO_2_ (116 mg/g) (Chen et al., 2016).

#### 4.2.1 Factors Affecting the Adsorption of Dyes by Templated Materials

The solution pH value is regarded as one of the most important factors affecting the adsorption of organic dyes. In general, the adsorption of anionic dyes increases with decreasing pH, while the opposite is true for cationic dyes. For anionic dyes, competition between OH^-^ and anionic dyes for adsorption is intense in the high pH range, while at low pH, the hydroxyl and carboxyl groups will be protonated to form -OH+ 2 groups, strengthening the electrostatic gravitational force between the adsorption site and the anionic dyes. For example, poly (acrylamide-acrylic acid-dimethylaminoethyl methacrylate) P(AAm-AA-DMAEMA) resin is effective for the adsorption of indigo carmine and eriochrome black-T anionic dyes, and the property of adsorption decreases with increasing pH (Abdel-Aziz and Siyam, 2011). The adsorption of methylene blue and crystalline violet by the MWCNTs@carbon with negative surface charge was low in acidic solutions and increased significantly with increasing pH (Liu et al., 2015).

However, unlike the templated materials for the adsorption of heavy metal ions, the templated materials for the adsorption of dyes are either endothermic or exothermic processes, resulting in different trends in their temperature dependence. For example, as the temperature increases, the adsorption of amorphous carbon nanotubes (ACNTs) increases (Zhong et al., 2019) while the adsorption of P(AAm-AA-DMAEMA) resin decreases (Abdel-Aziz and Siyam, 2011).

As can be seen from Table 4, the organic dye molecules almost always conform to the quasi-secondary kinetic model on the template materials. Unlike the adsorption of heavy metal ions, while the adsorption isotherms of most of the template materials conform to the Langmuir model, a few conform to the Freundlich model, indicating that for the adsorption of dyes, in addition to monolayer adsorption, multilayer adsorption can occur within the pore channels of a small number of template materials.

**TABLE 4:** Properties of various template materials and their ability to adsorb organic pollutant.

Sample	Template agent	Surface area (m^2^/g)	Adsorbate	Adsorption capacity (mg/g)	Equilibrium time (min)	pH	Kinetics	Isotherms	Cycle numbers	References
DP Mn_2_O_3_-carbon-PVP	Carbon spheres	37	Congo Red	126	20	—	Pseudo second order	Langmuir	4	Shao et al. (2017)
AHS	CaMg(CO_3_)_2_	318	Congo Red	690	120	5–6	Pseudo second order	Langmuir	—	Tian et al. (2016)
MWCNTs@carbon	MWCNTs	—	Methylene Blue	298	120	8–9	Pseudo second order	Langmuir	—	Liu et al. (2015)
			Crystal Violet	228						
CNTs	Pal	887	Congo Red	468	30	1–13	Pseudo second order	Langmuir	—	Zhong et al. (2019)
			Methyl Orange	253						
CMK-3	CMK	1420	Bisphenol-A	474	20	6	Pseudo second order	Freundlich	—	Libbrecht et al. (2015)
TNC	MCM-22	660	Amoxicillin	69	—	—	—	Langmuir	—	Barrera et al. (2014)
			Ethinylestradiol	61						
NMCS	SiO_2_	789	Methyl Orange	352	200	2	Pseudo second order	Langmuir	5	Jiao et al. (2017)
3D-HPGF	PMMA	486	Methylene Blue	70	25	—	Pseudo second order	Langmuir	5	Liu et al. (2018)
P (AAm-AA-DMAEMA)	PAAm	—	Indigo Carmine	—	120	2.5	Pseudo second order	Freundlich	—	Abdel-Aziz and Siyam, (2011)
			Eriochrome Black-T	—						
SMC	SMC	476	Bisphenol-A	154	60	1–7	Pseudo second order	Freundlich	—	Libbrecht et al. (2015)
S-2	Rape pollen	41	Congo Red	97	120	—	Pseudo second order	Langmuir	—	Zhao et al. (2017)
OMCs	F127	424	Rhodamine B	—	240	5	Pseudo second order	—	—	Cao et al. (2014)
C-V-50-T-50-600	PEO-PBO-PEO	810	Lysozyme	446	—	—	—	Langmuir	—	Wickramaratne and Jaroniec (2013)
NiCo-LDH	Ni(NO_3_)_2_	267	Congo red	909	80	—	Pseudo second order	Langmuir	5	Hu et al. (2019)

### 4.2.2 Mechanisms of Dye Adsorption

The excellent performance of the templated materials for dyes is partly attributed to the porous structure and high specific surface area. The porous structure and high specific surface area facilitate the exposure of more active sites, thus promoting the diffusion of dye molecules, that is, the efficient adsorption of dyes through adsorption and the filling of pore channels. Dual-porous Mn_2_O_3_ (DP Mn_2_O_3_-carbon-PVP) cubes have a specific surface area of 37 m^2^/g and the mesopore size of 24 nm, higher than that of the Mn_2_O_3_-Ncarbon prepared without polyvinylpyrrolidone (PVP) (22 m^2^/g, 23 nm) and without carbon sphere templates (15 m^2^/g, 4 nm). Comparing their sorption rates to Congo Red, DP Mn_2_O_3_-carbon-PVP had a sorption capacity of 126 mg/g, but 64 mg/g and 112 mg/g for Mn_2_O_3_-Ncarbon and Mn_2_O_3_-carbon (Shao et al., 2017). ACNTs have a high specific surface area of 877.09 m^2^/g, which is much higher than that of raw Pal (181.14 m^2^/g), H-Pal (157.50 m^2^/g), Pal@C (103.41 m^2^/g). The maximum adsorption of Congo Red by ACNTs (358 mg/g) was more than 20-fold times that of raw Pal, and nearly 14-fold higher than that of H-Pal and Pal@C (Zhong et al., 2019; Table 4).

In addition to the structure of the material, electrostatic interactions and π-π electron coupling interactions are also important mechanisms for the adsorption of dyes. For instance, the adsorption processes of nitrogen-doped mesoporous carbon spheres (NMCS) on methyl orange (Jiao et al., 2017) and ACTNs on Congo red (Zhong et al., 2019) are electrostatic attraction and π-π electron coupling, respectively, while the adsorption processes of hollow polydopamine microcapsules (H-PDAMCs) on methyl blue involve both electrostatic interaction and π-π stacking mechanisms (Feng et al., 2021). However, for materials with an intrinsic charge, the adsorption performance can be unsatisfactory due to electrostatic repulsion between the functional group of the dye and the functional group of the material. The introduction of template to adjust its surface charge can effectively improve this problem. For example, the large number of oxygen-containing functional groups such as -OH and -COOH generated on the surface of MWCNTs@carbon nanocables provides an abundance of active sites for positively charged dyes compared to the original MWCNTs (Zhang et al., 2017). The zero charge of Al_2_O_3_ is close to 9, using it as a template agent, the obtained alumina hollow microspheres (AHS) still have a high adsorption capacity for Congo Red when the initial solution pH is beyond 10 (Tian et al., 2016; Table 4).

## 5 Conclusions and Perspectives

Based on the properties and spatial domain-limiting capability of templates, this study introduces the types of templates and their synthetic routes, and the regulation behavior of materials by template method and its application in the adsorption of typical pollutants in wastewater has been reviewed. In comparison, hard templates are more stable and allow for precise regulation of material size and structure, but hard templates are often difficult to remove and can easily cause structural damage to the raw material after removal, while soft templates are easy to construct and have relatively mild experimental conditions and allow for the synthesis of different materials with different morphologies, but soft templates are less stable and less efficient. The behavior of the template method in modulating the morphological structure of the materials was further discussed and it was found that the adsorbents prepared by the template method had a high specific surface area and abundant pores and were able to successfully construct pore channel structures. The type and concentration of the template agent are found to be the most crucial factors affecting the morphology of the final product. Therefore, in practice, the most suitable template should be selected according to the performance requirements of the target product and the reaction conditions.

The large specific surface area and porous structure of the templated materials offer the possibility of efficient adsorption of heavy metal ions and dyes. The adsorption mechanism for heavy metal ions includes ion exchange, electrostatic interaction, and complexation, while electrostatic interaction, π-π electron stacking, and hydrogen bonding are possible for dyes. The application of templated materials as effective adsorbents in wastewater and their adsorption mechanisms are still at the research stage. Moreover, during the preparation process of templated materials, the removal of the template may lead to the collapse of the pore structure. Optimization of the textural properties and attention to the removal conditions are desired to ensure the effective diffusion of pollutants within the adsorbent.

Templated materials have good application prospects in removing pollution in wastewater. However, templated materials have been less studied for contaminants such as nutrients, biomolecules, and complex pollutants, and further research into the regeneration and desorption properties of adsorbents is required to achieve large-scale commercial use of templated materials.

## References

[B1] Abdel-Aziz H. M. Siyam T. (2011). Radiation Synthesis of Poly(Acrylamide-Acrylic Acid-Dimethylaminoethyl Methacrylate) Resin and its Use for Binding of Some Anionic Dyes. Water Air Soil Pollut. 218 (1), 165–174. 10.1007/s11270-010-0632-5

[B2] Abo-Zahra S. F. Abdelmonem I. M. Siyam T. E. El-Masry A. M. Abdel-Aziz H. M. (2021). Radiation Synthesis of Polyacrylamide/functionalized Multiwalled Carbon Nanotubes Composites for the Adsorption of Cu(II) Metal Ions from Aqueous Solution. Polym. Bull. 10.1007/s00289-021-03726-6

[B3] Ahmad R. Kim J. Kim J. Kim J. (2017). Nanostructured Ceramic Photocatalytic Membrane Modified with a Polymer Template for Textile Wastewater Treatment. Appl. Sci. 7 (12), 1284. 10.3390/app7121284

[B4] Alexandratos S. D. (2007). New Polymer-Supported Ion-Complexing Agents: Design, Preparation and Metal Ion Affinities of Immobilized Ligands. J. Hazard. Mater. 139 (3), 467–470. 10.1016/j.jhazmat.2006.02.042 16762497

[B5] Amali A. J. Sun J.-K. Xu Q. (2014). From Assembled Metal-Organic Framework Nanoparticles to Hierarchically Porous Carbon for Electrochemical Energy Storage. Chem. Commun. 50 (13), 1519–1522. 10.1039/c3cc48112c 24317277

[B6] Awual M. R. Hasan M. M. (2014). A Novel fine-tuning Mesoporous Adsorbent for Simultaneous Lead(II) Detection and Removal from Wastewater. Sensors Actuators B: Chem. 202, 395–403. 10.1016/j.snb.2014.05.103

[B7] Bao Y. Shi C. Wang T. Li X. Ma J. (2016). Recent Progress in Hollow Silica: Template Synthesis, Morphologies and Applications. Microporous Mesoporous Mater. 227. 10.1016/j.micromeso.2016.02.040

[B8] Barrera D. Villarroel-Rocha J. Tara J. C. Basaldella E. I. Sapag K. (2014). Synthesis and Textural Characterization of a Templated Nanoporous Carbon from MCM-22 Zeolite and its Use as Adsorbent of Amoxicillin and Ethinylestradiol. Adsorption 20 (8), 967–976. 10.1007/s10450-014-9640-x

[B9] Bo Z. Jing W. (2007). Pore Diameter Adjustment of Mesoporous Alumina with Different Templates. JOURNAL FUNCTIONAL MATERIALS DEVICES 13 (2), 150–154. 10.3969/j.issn.1007-4252.2007.02.010

[B10] Bourret G. R. Lennox R. B. (2010). 1D Cu(OH)2 Nanomaterial Synthesis Templated in Water Microdroplets. J. Am. Chem. Soc. 132 (19), 6657–6659. 10.1021/ja101579v 20411931

[B11] Brião G. V. Jahn S. L. Foletto E. L. Dotto G. L. (2017). Adsorption of crystal Violet Dye onto a Mesoporous ZSM-5 Zeolite Synthetized Using Chitin as Template. J. Colloid Interf. Sci. 508, 313–322. 10.1016/j.jcis.2017.08.070 28843109

[B12] Brown J. Richer R. Mercier L. (2000). One-step Synthesis of High Capacity Mesoporous Hg2+ Adsorbents by Non-ionic Surfactant Assembly. Microporous mesoporous Mater. 37 (1-2), 41–48. 10.1016/s1387-1811(99)00191-2

[B13] Cao Y. Zhu J. Z. Ding Y. Han G. Fan R. L. Fu H. Q. (2014). Pore Size Control of Ordered Mesoporous Carbons and Adsorption Performance of Dye Molecules. Amm 548-549, 38–42. 10.4028/www.scientific.net/AMM.548-549.38

[B14] Chen D. Zeng Z. Zeng Y. Zhang F. Wang M. (2016). Removal of Methylene Blue and Mechanism on Magnetic γ-Fe2O3/SiO2 Nanocomposite from Aqueous Solution. Water Resour. Industry 15, 1–13. 10.1016/j.wri.2016.05.003

[B15] Cui H. Qian Y. Li Q. Zhang Q. Zhai J. (2012). Adsorption of Aqueous Hg(II) by a Polyaniline/attapulgite Composite. Chem. Eng. J. 211-212, 216–223. 10.1016/j.cej.2012.09.057

[B16] Dai J. Ren F. Tao C. (2012). Adsorption Behavior of Fe(II) and Fe(III) Ions on Thiourea Cross-Linked Chitosan with Fe(III) as Template. Molecules 17 (4), 4388–4399. 10.3390/molecules17044388 22495549 PMC6269078

[B17] Demirbaş A. (2005). Adsorption of Cr (III) and Cr (VI) Ions from Aqueous Solutions on to Modified Lignin. Energ. Sourc. 27 (15), 1449–1455.

[B18] Deng Y. Yu T. Wan Y. Shi Y. Meng Y. Gu D. (2007). Ordered Mesoporous Silicas and Carbons with Large Accessible Pores Templated from Amphiphilic Diblock Copolymer Poly(ethylene Oxide)-B-Polystyrene. J. Am. Chem. Soc. 129 (6), 1690–1697. 10.1021/ja067379v 17243685

[B19] Ekinci E. Budinova T. Yardim F. Petrov N. Razvigorova M. Minkova V. (2002). Removal of Mercury Ion from Aqueous Solution by Activated Carbons Obtained from Biomass and Coals. Fuel Process. Techn. 77-78, 437–443. 10.1016/S0378-3820(02)00065-6

[B20] El-Hankari S. Aguilera-Sigalat J. Bradshaw D. (2016). Surfactant-assisted ZnO Processing as a Versatile Route to ZIF Composites and Hollow Architectures with Enhanced Dye Adsorption. J. Mater. Chem. A. 4 (35), 13509–13518. 10.1039/c6ta05893k

[B21] El-Sayed G. O. (2011). Removal of Methylene Blue and crystal Violet from Aqueous Solutions by palm Kernel Fiber. Desalination 272 (1-3), 225–232. 10.1016/j.desal.2011.01.025

[B22] Fan X. Zhang Z. Li G. Rowson N. A. (2004). Attachment of Solid Particles to Air Bubbles in Surfactant-free Aqueous Solutions. Chem. Eng. Sci. 59 (13), 2639–2645. 10.1016/j.ces.2004.04.001

[B23] Feng M. Yu S. Wu P. Wang Z. Liu S. Fu J. (2021). Rapid, High-Efficient and Selective Removal of Cationic Dyes from Wastewater Using Hollow Polydopamine Microcapsules: Isotherm, Kinetics, Thermodynamics and Mechanism. Appl. Surf. Sci. 542, 148633. 10.1016/j.apsusc.2020.148633

[B24] Fu J. Chen Z. Wang M. Liu S. Zhang J. Zhang J. (2015). Adsorption of Methylene Blue by a High-Efficiency Adsorbent (Polydopamine Microspheres): Kinetics, Isotherm, Thermodynamics and Mechanism Analysis. Chem. Eng. J. 259, 53–61. 10.1016/j.cej.2014.07.101

[B25] Fuertes A. B. Lota G. Centeno T. A. Frackowiak E. (2005). Templated Mesoporous Carbons for Supercapacitor Application. Electrochimica Acta 50 (14), 2799–2805. 10.1016/j.electacta.2004.11.027

[B26] Gao X. Lu F. Dong B. Liu Y. Gao Y. Zheng L. (2015). Facile Synthesis of Gold and Gold-Based alloy Nanowire Networks Using Wormlike Micelles as Soft Templates. Chem. Commun. 51 (5), 843–846. 10.1039/c4cc08549c 25426506

[B27] Han X. Tian P. Pang H. Song Q. Ning G. Yu Y. (2014). Facile Synthesis of Magnetic Hierarchical MgO-MgFe2O4 Composites and Their Adsorption Performance towards Congo Red. RSC Adv. 4 (53), 28119–28125. 10.1039/c4ra02313g

[B28] Hu H. Liu J. Xu Z. Zhang L. Cheng B. Ho W. (2019). Hierarchical Porous Ni/Co-LDH Hollow Dodecahedron with Excellent Adsorption Property for Congo Red and Cr(VI) Ions. Appl. Surf. Sci. 478, 981–990. 10.1016/j.apsusc.2019.02.008

[B29] Huang Y. Zeng X. Guo L. Lan J. Zhang L. Cao D. (2018). Heavy Metal Ion Removal of Wastewater by Zeolite-Imidazolate Frameworks. Separat. Purif. Techn. 194, 462–469. 10.1016/j.seppur.2017.11.068

[B30] Jiang Z. Sun H. Qin Z. Jiao X. Chen D. (2012). Synthesis of Novel ZnS Nanocages Utilizing ZIF-8 Polyhedral Template. Chem. Commun. 48 (30), 3620–3622. 10.1039/c2cc00004k 22396953

[B31] Jiao K. Zhang B. Yue B. Ren Y. Liu S. Yan S. (2005). Growth of Porous Single-crystal Cr2O3 in a 3-D Mesopore System. Chem. Commun. 45, 5618–5620. 10.1039/b512080b 16292367

[B32] Jiao Y. Xu L. Sun H. Deng Y. Zhang T. Liu G. (2017). Synthesis of Benzxazine-Based Nitrogen-Doped Mesoporous Carbon Spheres for Methyl orange Dye Adsorption. J. Porous Mater. 24 (6), 1565–1574. 10.1007/s10934-017-0396-z

[B33] Jun S. Joo S. H. Ryoo R. Kruk M. Jaroniec M. Liu Z. (2000). Synthesis of New, Nanoporous Carbon with Hexagonally Ordered Mesostructure. J. Am. Chem. Soc. 122 (43), 10712–10713. 10.1021/ja002261e

[B34] Kim T.-W. Ryoo R. Gierszal K. P. Jaroniec M. Solovyov L. A. Sakamoto Y. (2005). Characterization of Mesoporous Carbons Synthesized with SBA-16 Silica Template. J. Mater. Chem. 15 (15), 1560. 10.1039/b417804a

[B35] Kruk M. Jaroniec M. Ryoo R. Joo S. H. (2000). Characterization of Ordered Mesoporous Carbons Synthesized Using MCM-48 Silicas as Templates. J. Phys. Chem. B 104 (33), 7960–7968. 10.1021/jp000861u

[B36] Kuroki A. Hiroto M. Urushihara Y. Horikawa T. Sotowa K.-I. Alcántara Avila J. R. (2019). Adsorption Mechanism of Metal Ions on Activated Carbon. Adsorption 25 (6), 1251–1258. 10.1007/s10450-019-00069-7

[B37] Lei S. Yu F. Yang L. Shuaiguo Z. Mengmeng W. Jie M. (2018). Impacts of Different Templates on the Morphologies and Sulfuration Performances of Nano Ferric Oxides. CHEMICAL INDUSTRY ENGINEERING PROGRESS 37 (5), 1831–1836. 10.16085/j.issn.1000-6613.2017-1340

[B38] Li N. Bai R. Liu C. (2005). Enhanced and Selective Adsorption of Mercury Ions on Chitosan Beads Grafted with Polyacrylamide via Surface-Initiated Atom Transfer Radical Polymerization. Langmuir 21 (25), 11780–11787. 10.1021/la051551b 16316114

[B39] Li Y. Liu C. Xie Y. Li X. Fan X. Yuan L. (2013). Single-molecule Observation of the K+-induced Switching of Valinomycin within a Template Network. Chem. Commun. 49 (79), 9021–9023. 10.1039/c3cc44978e 23977669

[B40] Li Z. Shao M. Zhou L. Zhang R. Zhang C. Wei M. (2016). Directed Growth of Metal-Organic Frameworks and Their Derived Carbon-Based Network for Efficient Electrocatalytic Oxygen Reduction. Adv. Mater. 28 (12), 2337–2344. 10.1002/adma.201505086 26808408

[B41] Libbrecht W. Vandaele K. De Buysser K. Verberckmoes A. Thybaut J. Poelman H. (2015). Tuning the Pore Geometry of Ordered Mesoporous Carbons for Enhanced Adsorption of Bisphenol-A. Materials 8 (4), 1652–1665. 10.3390/ma8041652 28788023 PMC5507042

[B42] Libbrecht W. Verberckmoes A. Thybaut J. W. Van Der Voort P. De Clercq J. (2017). Soft Templated Mesoporous Carbons: Tuning the Porosity for the Adsorption of Large Organic Pollutants. Carbon 116, 528–546. 10.1016/j.carbon.2017.02.016

[B43] Liu D. Hu Y.-Y. Hu Y.-Y. Zeng C. Qu D.-Y. (2016a). Soft-Templated Ordered Mesoporous Carbon Materials: Synthesis, Structural Modification and Functionalization. Acta Physico-Chimica Sinica 32 (12), 2826–2840. 10.3866/pku.Whxb201609141

[B44] Liu J. Ge X. Ye X. Wang G. Zhang H. Zhou H. (2016b). 3D Graphene/δ-MnO2 Aerogels for Highly Efficient and Reversible Removal of Heavy Metal Ions. J. Mater. Chem. A. 4 (5), 1970–1979. 10.1039/c5ta08106h

[B45] Liu L. Liu S. Peng H. Yang Z. Zhao L. Tang A. (2020). Surface Charge of Mesoporous Calcium Silicate and its Adsorption Characteristics for Heavy Metal Ions. Solid State. Sci. 99, 106072. 10.1016/j.solidstatesciences.2019.106072

[B46] Liu W. Jiang X. Chen X. (2015). Synthesis and Utilization of a Novel Carbon Nanotubes Supported Nanocables for the Adsorption of Dyes from Aqueous Solutions. J. Solid State. Chem. 229, 342–349. 10.1016/j.jssc.2015.06.026

[B47] Liu X. Wang R. He Y. Ni Z. Su N. Guo R. (2019). Construction of Alternating Layered Quasi-Three-Dimensional Electrode Ag NWs/CoO for Water Splitting: A Discussion of Catalytic Mechanism. Electrochimica Acta 317, 468–477. 10.1016/j.electacta.2019.06.029

[B104] Liu Y. Goebl J. Yin Y. (2013). Templated Synthesis of Nanostructured Materials. Chem. Soc. Rev. 42 (7), 2610–2653. 10.1039/C2CS35369E 23093173

[B48] Liu Y. Hu R. Zhang Z. (2018). A Facile Colloidal crystal Templating Method to Produce Three-Dimensional Hierarchical Porous Graphene-Fe3O4 Nanocomposite for the Removal of Dyes from Aqueous Solution. J. Porous Mater. 26 (1), 271–280. 10.1007/s10934-018-0653-9

[B49] Liu Y. Qiao L. Wang A. Li Y. Zhao L. Du K. (2021). Tentacle-type Poly(hydroxamic Acid)-Modified Macroporous Cellulose Beads: Synthesis, Characterization, and Application for Heavy Metal Ions Adsorption. J. Chromatogr. A 1645, 462098. 10.1016/j.chroma.2021.462098 33848662

[B50] Lü Q. F. Huang M. R. Li X. G. (2007). Synthesis and Heavy‐metal‐ion Sorption of Pure Sulfophenylenediamine Copolymer Nanoparticles with Intrinsic Conductivity and Stability. Chemistry–A Eur. J. 13 (21), 6009–6018. 10.1002/chem.200700233 17487909

[B51] Ma Y. Zhuang Z. Ma M. Yang Y. Li W. lin J. (2019). Solid Polyaniline Dendrites Consisting of High Aspect Ratio Branches Self-Assembled Using Sodium Lauryl Sulfonate as Soft Templates: Synthesis and Electrochemical Performance. Polymer 182, 121808. 10.1016/j.polymer.2019.121808

[B52] Ma Z. Gao J. Su W. Zhang Q. Wang X. (2020). Effect of Template Agent on Synthetic Aluminosilicate Mesoporous Material. MATEIALS SCIENCE TECHNOLOGY 28 (06), 49–55. 10.11951/j.issn.1005-0299.20190015

[B53] Madima N. Mishra S. B. Inamuddin I. Mishra A. K. (2020). Carbon-based Nanomaterials for Remediation of Organic and Inorganic Pollutants from Wastewater. A Review. Environ. Chem. Lett. 18 (4), 1169–1191. 10.1007/s10311-020-01001-0

[B54] Manyangadze M. Chikuruwo N. M. H. Narsaiah T. B. Chakra C. S. Charis G. Danha G. (2020). Adsorption of lead Ions from Wastewater Using Nano Silica Spheres Synthesized on Calcium Carbonate Templates. Heliyon 6 (11), e05309. 10.1016/j.heliyon.2020.e05309 33204869 PMC7649267

[B55] Marciniak M. Goscianska J. Frankowski M. Pietrzak R. (2019). Optimal Synthesis of Oxidized Mesoporous Carbons for the Adsorption of Heavy Metal Ions. J. Mol. Liquids 276, 630–637. 10.1016/j.molliq.2018.12.042

[B56] Marrakchi F. Ahmed M. J. Khanday W. A. Asif M. Hameed B. H. (2017). Mesoporous-activated Carbon Prepared from Chitosan Flakes via Single-step Sodium Hydroxide Activation for the Adsorption of Methylene Blue. Int. J. Biol. Macromolecules 98, 233–239. 10.1016/j.ijbiomac.2017.01.119 28147233

[B57] Meng C. Zhikun W. Qiang L. Chunling L. Shuangqing S. Songqing H. (2018). Preparation of Amino-Functionalized Fe3O4@mSiO2 Core-Shell Magnetic Nanoparticles and Their Application for Aqueous Fe3+ Removal. J. Hazard. Mater. 341, 198–206. 10.1016/j.jhazmat.2017.07.062 28780434

[B58] Miao Y. Zhai Z. He J. Li B. Li J. Wang J. (2010). Synthesis, Characterizations and Photocatalytic Studies of Mesoporous Titania Prepared by Using Four Plant Skins as Templates. Mater. Sci. Eng. C 30 (6), 839–846. 10.1016/j.msec.2010.03.020

[B59] Mohajeri M. Akbarpour H. Karimkhani V. (2017). Synthesis of Highly Ordered Carbon Nanotubes/nanoporous Anodic Alumina Composite Membrane and Potential Application in Heavy Metal Ions Removal from Industrial Wastewater. Mater. Today Proc. 4 (3), 4906–4911. 10.1016/j.matpr.2017.04.094

[B60] Pang M. Cairns A. J. Liu Y. Belmabkhout Y. Zeng H. C. Eddaoudi M. (2013a). Synthesis and Integration of Fe-Soc-MOF Cubes into Colloidosomes via a Single-step Emulsion-Based Approach. J. Am. Chem. Soc. 135 (28), 10234–10237. 10.1021/ja403994u 23822718

[B61] Pang X. Zhao L. Han W. Xin X. Lin Z. (2013b). A General and Robust Strategy for the Synthesis of Nearly Monodisperse Colloidal Nanocrystals. Nat. Nanotech 8 (6), 426–431. 10.1038/nnano.2013.85 23728076

[B62] Petkovich N. D. Stein A. (2013). Controlling Macro- and Mesostructures with Hierarchical Porosity through Combined Hard and Soft Templating. Chem. Soc. Rev. 42 (9), 3721–3739. 10.1039/c2cs35308c 23072972

[B63] Pi Y. Li X. Xia Q. Wu J. Li Y. Xiao J. (2018). Adsorptive and Photocatalytic Removal of Persistent Organic Pollutants (POPs) in Water by Metal-Organic Frameworks (MOFs). Chem. Eng. J. 337, 351–371. 10.1016/j.cej.2017.12.092

[B64] Pongkitdachoti U. Unob F. (2018). Simultaneous Adsorption of Silver Nanoparticles and Silver Ions on Large Pore Mesoporous Silica. J. Environ. Chem. Eng. 6 (1), 596–603. 10.1016/j.jece.2017.12.046

[B65] Ren C. Ding X. Li W. Wu H. Yang H. (2017). Highly Efficient Adsorption of Heavy Metals onto Novel Magnetic Porous Composites Modified with Amino Groups. J. Chem. Eng. Data 62 (6), 1865–1875. 10.1021/acs.jced.7b00198

[B66] Roggenbuck J. Koch G. Tiemann M. (2006). Synthesis of Mesoporous Magnesium Oxide by CMK-3 Carbon Structure Replication. Chem. Mater. 18 (17), 4151–4156. 10.1021/cm060740s

[B67] Salunkhe R. R. Young C. Tang J. Takei T. Ide Y. Kobayashi N. (2016). A High-Performance Supercapacitor Cell Based on ZIF-8-Derived Nanoporous Carbon Using an Organic Electrolyte. Chem. Commun. 52 (26), 4764–4767. 10.1039/c6cc00413j 26928244

[B68] Sarma G. K. Sen Gupta S. Bhattacharyya K. G. (2019). Nanomaterials as Versatile Adsorbents for Heavy Metal Ions in Water: a Review. Environ. Sci. Pollut. Res. 26 (7), 6245–6278. 10.1007/s11356-018-04093-y 30623336

[B69] Savic S. Vojisavljevic K. Počuča-Nešić M. Zivojevic K. Mladenovic M. Knezevic N. (2018). Hard Template Synthesis of Nanomaterials Based on Mesoporous Silica. Metall. Mater. Eng. 24 (4). 10.30544/400

[B70] Schüth F. (2003). Endo- and Exotemplating to Create High-Surface-Area Inorganic Materials. Angew. Chem. Int. Ed. 42 (31), 3604–3622. 10.1002/anie.200300593 12916030

[B71] Senberber F. T. Yildirim M. Mermer N. K. Derun E. M. (2017). Adsorption of Cr(III) from Aqueous Solution Using Borax Sludge. ACSi 64 (3), 654–660. 10.17344/acsi.2017.3534 28862303

[B72] Shao Y. Ren B. Jiang H. Zhou B. Lv L. Ren J. (2017). Dual-porosity Mn2O3 Cubes for Highly Efficient Dye Adsorption. J. Hazard. Mater. 333, 222–231. 10.1016/j.jhazmat.2017.03.014 28359038

[B73] Shen K. Zhang L. Chen X. Liu L. Zhang D. Han Y. (2018). Ordered Macro-Microporous Metal-Organic Framework Single Crystals. Science 359 (6372), 206–210. 10.1126/science.aao3403 29326271

[B74] Singh J. Sharma M. Basu S. (2018). Heavy Metal Ions Adsorption and Photodegradation of Remazol Black XP by Iron Oxide/silica Monoliths: Kinetic and Equilibrium Modelling. Adv. Powder Techn. 29 (9), 2268–2279. 10.1016/j.apt.2018.06.011

[B75] Sun S. Wang Q. Wang A. (2007). Adsorption Properties of Cu(II) Ions onto N-Succinyl-Chitosan and Crosslinked N-Succinyl-Chitosan Template Resin. Biochem. Eng. J. 36 (2), 131–138. 10.1016/j.bej.2007.02.010

[B76] Sun Y. Shao D. Chen C. Yang S. Wang X. (2013). Highly Efficient Enrichment of Radionuclides on Graphene Oxide-Supported Polyaniline. Environ. Sci. Technol. 47 (17), 9904–9910. 10.1021/es401174n 23902375

[B77] Sun Y. Wu Z.-Y. Wang X. Ding C. Cheng W. Yu S.-H. (2016). Macroscopic and Microscopic Investigation of U(VI) and Eu(III) Adsorption on Carbonaceous Nanofibers. Environ. Sci. Technol. 50 (8), 4459–4467. 10.1021/acs.est.6b00058 26998856

[B78] Teoh Y. P. Khan M. A. Choong T. S. Y. (2013). Kinetic and Isotherm Studies for lead Adsorption from Aqueous Phase on Carbon Coated Monolith. Chem. Eng. J. 217, 248–255. 10.1016/j.cej.2012.12.013

[B79] Thomas A. Goettmann F. (2008). Hard Templates for Soft Materials: Creating Nanostructured Organic Materials. Chem. Mater. 20 (3), 738–755. 10.1021/cm702126j

[B80] Tian J. Tian P. Pang H. Ning G. Bogale R. F. Cheng H. (2016). Fabrication Synthesis of Porous Al 2 O 3 Hollow Microspheres and its superior Adsorption Performance for Organic Dye. Microporous Mesoporous Mater. 223, 27–34. 10.1016/j.micromeso.2015.09.055

[B81] Wan Y. Zhao D. Y. (2007). On the Controllable Soft-Templating Approach to Mesoporous Silicates. Chem. Rev. 107 (7), 2821–2860. 10.1021/cr068020s 17580976

[B82] Wang B. Chen J. S. Wu H. B. Wang Z. Lou X. W. (2011a). Quasiemulsion-Templated Formation of α-Fe2O3 Hollow Spheres with Enhanced Lithium Storage Properties. J. Am. Chem. Soc. 133 (43), 17146–17148. 10.1021/ja208346s 21977903

[B83] Wang H. Qu Z. G. Zhang W. Zhang L. Q. (2016a). A Multi-Scale Porous Composite Adsorbent with Copper Benzene-1,3,5-Tricarboxylate Coating on Copper Foam. RSC Adv. 6 (58), 52888–52897. 10.1039/c6ra08622e

[B84] Wang J. Deng B. Chen H. Wang X. Zheng J. (2009). Removal of Aqueous Hg(II) by Polyaniline: Sorption Characteristics and Mechanisms. Environ. Sci. Technol. 43 (14), 5223–5228. 10.1021/es803710k 19708345

[B85] Wang L. Wang J. He C. Lyu W. Zhang W. Yan W. (2019a). Development of Rare Earth Element Doped Magnetic Biochars with Enhanced Phosphate Adsorption Performance. Colloids Surf. A: Physicochemical Eng. Aspects 561, 236–243. 10.1016/j.colsurfa.2018.10.082

[B86] Wang L. Wang J. Wang Z. Feng J. Li S. Yan W. (2019b). Synthesis of Ce-Doped Magnetic Biochar for Effective Sb(V) Removal: Performance and Mechanism. Powder Techn. 345, 501–508. 10.1016/j.powtec.2019.01.022

[B87] Wang L. Wang J. Wei Y. (2021). Facile Synthesis of Eggshell Biochar Beads for superior Aqueous Phosphate Adsorption with Potential Urine P-Recovery. Colloids Surf. A: Physicochemical Eng. Aspects 622, 126589. 10.1016/j.colsurfa.2021.126589

[B88] Wang L. Wang J. Yan W. He C. Shi Y. (2020a). MgFe2O4-biochar Based Lanthanum Alginate Beads for Advanced Phosphate Removal. Chem. Eng. J. 387, 123305. 10.1016/j.cej.2019.123305

[B89] Wang X. Zhong Y. Zhai T. Guo Y. Chen S. Ma Y. (2011b). Multishelled Co3O4-Fe3O4 Hollow Spheres with Even Magnetic Phase Distribution: Synthesis, Magnetic Properties and Their Application in Water Treatment. J. Mater. Chem. 21 (44), 17680–17687. 10.1039/c1jm13180j

[B90] Wang Y. Jiang Q. Jiang Q. Shang J.-K. Xu J. Li Y.-X. (2016b). Advances in the Synthesis of Mesoporous Carbon Nitride Materials. Acta Physico-Chimica Sinica 32 (8), 1913–1928. 10.3866/pku.Whxb201605052

[B91] Wang Y. Yang X. Jing X. Dai J. Dong M. Yan Y. (2020b). Adsorption of Phosphorus on Lanthanum Doped Carbon Films Guided by Self-Assembly of Cellulose Nanocrystalline. J. Mol. Liquids 319, 114148. 10.1016/j.molliq.2020.114148

[B92] Wei Y. Wang L. Wang J. (2022). Cerium Alginate Cross-Linking with Biochar Beads for Fast Fluoride Removal over a Wide pH Range. Colloids Surf. A: Physicochemical Eng. Aspects 636, 128161. 10.1016/j.colsurfa.2021.128161

[B93] Wickramaratne N. P. Jaroniec M. (2013). Phenolic Resin-Based Carbons with Ultra-large Mesopores Prepared in the Presence of Poly(ethylene Oxide)-Poly(butylene Oxide)-Poly(ethylene Oxide) Triblock Copolymer and Trimethyl Benzene. Carbon 51, 45–51. 10.1016/j.carbon.2012.08.007

[B94] Wu D. Xu F. Sun B. Fu R. He H. Matyjaszewski K. (2012). Design and Preparation of Porous Polymers. Chem. Rev. 112 (7), 3959–4015. 10.1021/cr200440z 22594539

[B95] Wu X.-J. Xu D. (2010). Soft Template Synthesis of Yolk/silica Shell Particles. Adv. Mater. 22 (13), 1516–1520. 10.1002/adma.200903879 20437501

[B96] Wu Y.-n. Li F. Zhu W. Cui J. Tao C.-a. Lin C. (2011). Metal-organic Frameworks with a Three-Dimensional Ordered Macroporous Structure: Dynamic Photonic Materials. Angew. Chem. Int. Ed. 50 (52), 12518–12522. 10.1002/anie.201104597 22006864

[B97] Xie Y. Kocaefe D. Chen C. Kocaefe Y. Seki S. (2016). Review of Research on Template Methods in Preparation of Nanomaterials. J. Nanomater. 2302595. 10.1155/2016/2302595

[B98] Xie Y. Song Y. Zhang Y. Xu L. Miao L. Peng C. (2018). Cu Metal-Organic Framework-Derived Cu Nanospheres@Porous Carbon/macroporous Carbon for Electrochemical Sensing Glucose. J. Alloys Comp. 757, 105–111. 10.1016/j.jallcom.2018.05.064

[B99] Yan C. Xue D. (2007). Polyhedral Construction of Hollow ZnO Microspheres by CO2 Bubble Templates. J. Alloys Comp. 431 (1-2), 241–245. 10.1016/j.jallcom.2006.05.064

[B100] Yan L. Li Q. Wang X. Song H. Chi H. Qiao Y. (2017). Synthesis and Absorption Performance of Acrylic Ester and Hollow Fiber MgO Nanoparticle Resin Composite. Polymer-Plastics Techn. Eng. 56 (17), 1857–1865. 10.1080/03602559.2017.1295310

[B101] Yang L. Li R. Zhang L. Jiang X. Wang R. Tian B. (2021). Preparation of Flower-like G-C_3N_4/Bi_2MoO_6 Microspheres and Their Photocatalytic Degradation of Simulated Dye Wastewater. Fine Chemicals 38 (5), 1030–1037.

[B102] Yang L. M. Wang Y. J. Sun Y. W. Luo G. S. Dai Y. Y. (2006). Synthesis of Micrometer-Sized Hard Silica Spheres with Uniform Mesopore Size and Textural Pores. J. Colloid Interf. Sci. 299 (2), 823–830. 10.1016/j.jcis.2006.02.043 16616179

[B103] Yang X. Yan L. Ma J. Bai Y. Shao L. (2019). Bioadhesion-inspired Surface Engineering Constructing Robust, Hydrophilic Membranes for Highly-Efficient Wastewater Remediation. J. Membr. Sci. 591, 117353. 10.1016/j.memsci.2019.117353

[B105] Yu G. Wang X. Liu J. Jiang P. You S. Ding N. (2021). Applications of Nanomaterials for Heavy Metal Removal from Water and Soil: A Review. Sustainability 13 (2), 713. 10.3390/su13020713

[B106] Yu L. Wang L. Xu W. Chen L. Fu M. Wu J. (2018). Adsorption of VOCs on Reduced Graphene Oxide. J. Environ. Sci. 67, 171–178. 10.1016/j.jes.2017.08.022 29778150

[B107] Yue W. Zhou W. (2007). Porous Crystals of Cubic Metal Oxides Templated by Cage-Containing Mesoporous Silica. J. Mater. Chem. 17 (47), 4947. 10.1039/b709076e

[B108] Yusuf M. M. Imai H. Hirashima H. (2003). Preparation of Mesoporous Titania by Templating with Polymer and Surfactant and its Characterization. J. sol-gel Sci. Technol. 28 (1), 97–104. 10.1023/a:1025645305557

[B109] Zanin E. Scapinello J. de Oliveira M. Rambo C. L. Franscescon F. Freitas L. (2017). Adsorption of Heavy Metals from Wastewater Graphic Industry Using Clinoptilolite Zeolite as Adsorbent. Process Saf. Environ. Prot. 105, 194–200. 10.1016/j.psep.2016.11.008

[B110] Zare E. N. Motahari A. Sillanpää M. (2018). Nanoadsorbents Based on Conducting Polymer Nanocomposites with Main Focus on Polyaniline and its Derivatives for Removal of Heavy Metal Ions/dyes: A Review. Environ. Res. 162, 173–195. 10.1016/j.envres.2017.12.025 29329014

[B111] Zhang C. Li X. Chen Z. Wen T. Huang S. Hayat T. (2017). Synthesis of Ordered Mesoporous Carbonaceous Materials and Their Highly Efficient Capture of Uranium from Solutions. Sci. China Chem. 61 (3), 281–293. 10.1007/s11426-017-9132-7

[B112] Zhang D. Zhang C.-l. Zhou P. (2011). Preparation of Porous Nano-Calcium Titanate Microspheres and its Adsorption Behavior for Heavy Metal Ion in Water. J. Hazard. Mater. 186 (2-3), 971–977. 10.1016/j.jhazmat.2010.11.096 21177018

[B113] Zhang L.-L. Song Y. Song Y. Li G.-D. Zhang S.-L. Shang Y.-S. (2015). ZSM-5 Zeolite with Micro-mesoporous Structures Synthesized Using Different Templates for Methanol to Propylene Reaction. Acta Physico-Chimica Sinica 31 (11), 2139–2150. 10.3866/pku.Whxb201509281

[B114] Zhang L. Tang A. Wang Z. Zhang M. Xu L. Zhu L. (2018). Preparation of Polydopamine/poly (Ethylene Glycol) Composite Nanocapsules with ZIF-8 Nanoparticles as Templates. J. Funct. Polym. 31 (6), 546–552. 10.14133/j.cnki.1008-9357.20180714001

[B115] Zhang Y. Sun H. Sadam H. Liu Y. Shao L. (2019). Supramolecular Chemistry Assisted Construction of Ultra-stable Solvent-Resistant Membranes for Angstrom-Sized Molecular Separation. Chem. Eng. J. 371, 535–543. 10.1016/j.cej.2019.04.096

[B116] Zhao J. Shao Q. Ge S. Zhang J. Lin J. Cao D. (2020b). Advances in Template Prepared Nano-Oxides and Their Applications: Polluted Water Treatment, Energy, Sensing and Biomedical Drug Delivery. Chem. Rec. 20 (7), 710–729. 10.1002/tcr.201900093 31944590

[B117] Zhao J. Ge S. Liu L. Shao Q. Mai X. Zhao C. X. (2017). Microwave Solvothermal Fabrication of Zirconia Hollow Microspheres with Different Morphologies Using Pollen Templates and Their Dye Adsorption Removal. Ind. Eng. Chem. Res. 57 (1), 231–241. 10.1021/acs.iecr.7b04000

[B118] Zhao J. Shao Q. Ge S. Zhang J. Lin J. Cao D. (2020a). Advances in Template Prepared Nano‐Oxides and Their Applications: Polluted Water Treatment, Energy, Sensing and Biomedical Drug Delivery. Chem. Rec. 20 (7), 710–729. 10.1002/tcr.201900093 31944590

[B119] Zhao Q. Gao Y. Bai X. Wu C. Xie Y. (2006). Facile Synthesis of SnO 2 Hollow Nanospheres and Applications in Gas Sensors and Electrocatalysts. Eur. J. Inorg. Chem. 2006 (8), 1643–1648. 10.1002/ejic.200500975

[B120] Zhao X. Yu X. Wang X. Lai S. Sun Y. Yang D. (2021). Recent Advances in Metal-Organic Frameworks for the Removal of Heavy Metal Oxoanions from Water. Chem. Eng. J. 407, 127221. 10.1016/j.cej.2020.127221

[B121] Zhao X. Yuan Y. Li P. Song Z. Ma C. Pan D. (2019). A Polyether Amine Modified Metal Organic Framework Enhanced the CO2 Adsorption Capacity of Room Temperature Porous Liquids. Chem. Commun. 55 (87), 13179–13182. 10.1039/c9cc07243h 31620709

[B122] Zheng X. Xie Y. Zhu L. Jiang X. Yan A. (2002). Formation of Vesicle-Templated CdSe Hollow Spheres in an Ultrasound-Induced Anionic Surfactant Solution. Ultrason. Sonochem. 9 (6), 311–316. 10.1016/s1350-4177(02)00086-x 12404796

[B123] Zhong L. Tang A. Yan P. Wang J. Wang Q. Wen X. (2019). Palygorskite-template Amorphous Carbon Nanotubes as a superior Adsorbent for Removal of Dyes from Aqueous Solutions. J. Colloid Interf. Sci. 537, 450–457. 10.1016/j.jcis.2018.11.016 30465979

[B124] Zhou X. Lai C. Huang D. Zeng G. Chen L. Qin L. (2018). Preparation of Water-Compatible Molecularly Imprinted Thiol-Functionalized Activated Titanium Dioxide: Selective Adsorption and Efficient Photodegradation of 2, 4-dinitrophenol in Aqueous Solution. J. Hazard. Mater. 346, 113–123. 10.1016/j.jhazmat.2017.12.032 29253750

[B125] Zhou Y. Zhang L. Cheng Z. (2015). Removal of Organic Pollutants from Aqueous Solution Using Agricultural Wastes: A Review. J. Mol. Liquids 212, 739–762. 10.1016/j.molliq.2015.10.023

[B126] Zhu S. Zhao N. Li J. Deng X. Sha J. He C. (2019). Hard-template Synthesis of Three-Dimensional Interconnected Carbon Networks: Rational Design, Hybridization and Energy-Related Applications. Nano Today 29, 100796. 10.1016/j.nantod.2019.100796

[B127] Zhu T. Chen J. S. Lou X. W. (2012). Highly Efficient Removal of Organic Dyes from Waste Water Using Hierarchical NiO Spheres with High Surface Area. J. Phys. Chem. C 116 (12), 6873–6878. 10.1021/jp300224s

[B128] Zhu X. Alexandratos S. D. (2005). Polystyrene-Supported Amines: Affinity for Mercury(II) as a Function of the Pendant Groups and the Hg(II) Counterion. Ind. Eng. Chem. Res. 44 (23), 8605–8610. 10.1021/ie048736i

